# Prediction of Inhibitory Activity against the MATE1 Transporter via Combined Fingerprint- and Physics-Based Machine Learning Models

**DOI:** 10.1021/acs.jcim.4c00921

**Published:** 2024-09-10

**Authors:** Koichi Handa, Shunta Sasaki, Satoshi Asano, Michiharu Kageyama, Takeshi Iijima, Andreas Bender

**Affiliations:** † Centre for Molecular Informatics, Department of Chemistry, University of Cambridge, Lensfield Road, Cambridge CB2 1EW, U.K.; ‡ Toxicology & DMPK Research Department, Teijin Institute for Bio-medical Research, Teijin Pharma Limited, 4-3-2 Asahigaoka, Hino-shi, Tokyo 191-8512, Japan; § Pharmaceutical Discovery Research Laboratories, Teijin Pharma Limited, Tokyo 191-8512, Japan; ∥ Institutul STAR-UBB, Universitatea Babes-Bolyai, Str. Mihail Kogălniceanu nr. 1, Cluj-Napoca 400084, Romania

## Abstract

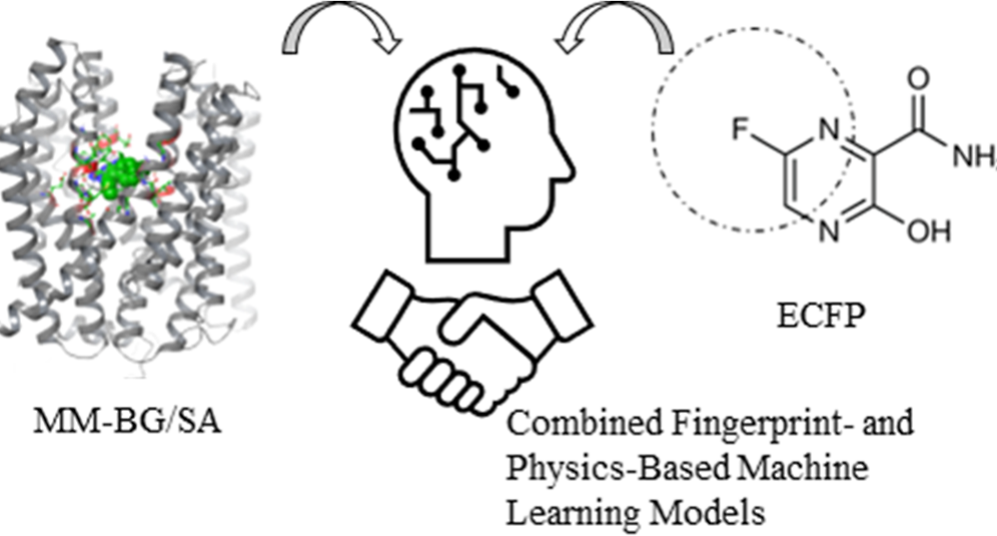

Renal secretion plays an important role in excretion of drug from the kidney. Two major transporters known to be highly involved in renal secretion are MATE1/2 K and OCT2, the former of which is highly related to drug–drug interactions. Among published in silico models for MATE inhibitors, a previous model obtained a ROC-AUC value of 0.78 using high throughput percentage inhibition data [*J. Med. Chem.***2013**, *56*(3), 781–795] which we aimed to improve upon here using a combined fingerprint and physics-based approach. To this end, we collected 225 publicly available compounds with pIC50 values against MATE1. Subsequently, on the one hand, we performed a physics-based approach using an Alpha-Fold protein structure, from which we obtained MM–GB/SA scores for those compounds. On the other hand, we built Random Forest (RF) and message passing neural network models using extended-connectivity fingerprints with radius 4 (ECFP4) and chemical structures as graphs, respectively, which also included MM–GB/SA scores as input variables. In a five-fold cross-validation with a separate test set, we found that the best predictivity for the hold-out test was observed in the RF model (including ECFP4 and MM–GB/SA data) with an ROC-AUC of 0.833 ± 0.036; while that of the MM–GB/SA regression model was 0.742. However, the MM–GB/SA model did not show a dependency of the performance on the particular chemical space being predicted. Additionally, via structural interaction fingerprint analysis, we identified interacting residues with inhibitor as identical for those with noninhibitors, including substrates, such as Gln49, Trp274, Tyr277, Tyr299, Ile303, and Tyr306. The similar binding modes are consistent with the observed similar IC50 value inhibitor when using different substrates experimentally, and practically, this can release the experimental scientists from bothering of selecting substrates for MATE1. Hence, we were able to build highly predictive classification models for MATE1 inhibitory activity with both ECFP4 and MM–GB/SA score as input features, which is fit-for-purpose for use in the drug discovery process.

## 1 Introduction

Renal excretion of drugs involves glomerular filtration, tubular secretion, and reabsorption processes.^1^ Compounds whose renal clearance is greater than glomerular filtration clearance will be subject to renal tubular secretion and hence can interact with transporters expressed on renal epithelial cells. Most cationic organic compounds are substrates for the organic cation transporter (OCT) 2, multidrug and toxin extrusion transporter (MATE) 1, and MATE2-K.^2^ Their renal excretion in vivo is largely mediated by OCT2, which is expressed on the apical side of renal epithelial cells, and MATE1, MATE2-K which are expressed on the basolateral side.^2^ It has been clinically reported that concomitant use of drugs which are also inhibitors of OCT2, MATE1, and MATE2-K results in drug–drug interactions (DDI) with decreased renal excretion. For example, renal clearance of metformin, which is a substrate for OCT2, MATE1, and MATE-2K, was decreased in the coadministration of cimetidine and pyrimethamine, which are inhibitors of those transporters.^3,4^ Regarding the mechanism of the clinical DDI between cimetidine and metformin, it has been hypothesized mechanistically that MATE1 and MATE2-K play major roles for DDIs in the kidney because of the lower *K*_*i*_ values for MATE1 and MATE2-K than OCT2.^5^ This was also supported by physiologically based pharmacokinetic modeling.^6^ MATE1 that is significantly more highly expressed than MATE2-K is considered to have the similar potency of transportability with MATE2-K, and we have limited information on specific substrates in MATE2-K, suggesting that MATE1 plays a major role in the renal DDI of drugs.^7−9^ The current DDI guidelines (of the United States,^10^ Europe,^11^ and Japan^12^) list MATE1 as a transporter to be evaluated, suggesting that MATE1 is a clinically relevant transporter in terms of clinical DDI risk assessment. Consequently, a predictive method to prospectively evaluate the inhibitory potential of MATE1 in the early drug discovery stages is needed to evaluate new molecular entities with respect to this clinically relevant end point.

As for related work which aimed to predict inhibitory activity against MATE1, there are five publications we should mention here. In 2012, a Bayesian classification model was published using 46 compounds with functional connectivity fingerprint 6 and physicochemical properties. Since the ROC-AUC in two-fold cross validation (CV) was 0.82, this model was considered to have high predictivity.^13^ However, the number of compounds was limited for the model to be utilized in practical drug discovery projects from the viewpoint of chemical space coverage. In 2013, a larger data set of 910 compounds was used to predict percentage inhibition at 20 μM concentration, with 50% inhibition used as the threshold for a binary classification model^14^ based on random forest (RF) with 21 Dragon descriptors.^15^ The ROC-AUC by multiple external data sets was 0.78, and this model hence also had high numerical predictivity leading the highest model’s effectiveness so far. In 2015, a pharmacophore model was constructed using docked poses obtained from GLIDE.^16^ The data set was the same as in the preceding study,^14^ and 42 inhibitors were used to construct the pharmacophore model and tested by another 42 inhibitors and 398 noninhibitors. Since the best balanced accuracy of this model was 0.59, the predictivity was numerically moderate and not so effective; however, this research for the first time utilized structural information in MATE1 modeling.^17^ The most recent models also used structural information: in one study performed in early 2021, although there was no construction of predictive models, the authors built a homology model of hMATE1 from PDB entry 3MKT (the multidrug transport protein NorM from *Vibrio cholerae*).^18^ In subsequent work performed in late 2021, Alphafold2 was used to build a MATE1 protein structure, which resulted in a pharmacophore classification model with an accuracy of 0.51,^19^ which still leaves some room for improvement. In this research, the authors also built a machine learning classification model using Neural Networks (with an accuracy of 0.83) and support vector machines (with an accuracy 0.75). Although the accuracies were relatively high numerically, the thresholds of active/inactive were derived from percentage inhibition values (here of less than 10% and more than 50% inhibition), which is not a quantitative inhibition value, and performance values were likely inflated due to the range of activities in between the thresholds not considered in the model. Also, the compounds used for modeling were limited to 58 tyrosine kinase inhibitors, which would not allow for model application across wider chemical space. Consequently, this model was not always effective. Based on these related studies, all of them are classification models; however, in practice often two-class models are not sufficient for decision making, which also agrees with regulatory guidelines.^10−12^ To sum up these related studies, on the one hand, we can find the difficulty of the construction of regression models, assuming that it is due to the shortage of reliable experimental data. On the other hand, the progress of utilizing structural information can be seen as a promising method for the prediction of inhibitory activity of MATE1.

Based on the above related work, in this study we aimed to identify a new approach, based jointly on ligand features and ligand–protein interaction scores. While docking scores have been used frequently in particular in high-throughput settings, correlations with quantitative activity are not always a given,^20,21^ which is why in this work instead of docking we used molecular mechanics with generalized born and surface area solvation (MM–GB/SA) scores as a more accurate scoring function of binding affinity.^22−26^ MM–GB/SA includes the interaction between not only ligand and receptor but also receptor and solvent (water) when calculating enthalpy (Δ*H*) and also entropy (Δ*S*).^27,28^ Practically MM–GB/SA is often used in the drug discovery process where better estimates of affinity are needed, which is also why we used it in the current work.

In this study considering both the importance of the exact values from IC50 or *K*_*i*_ data and the difficulty of constructing the regression model, we hence built in silico models for the classification with the practical threshold of the inhibitory activity (10 μmol/L) against MATE1 using three different types of information to explore their information content, namely, an MM–GB/SA model by itself, a machine learning model based on ligand chemical descriptors, and finally a machine learning model utilizing both ligand chemical descriptors and MM–GB/SA scores.

## 2 Materials and Methods

**Figure 1:**
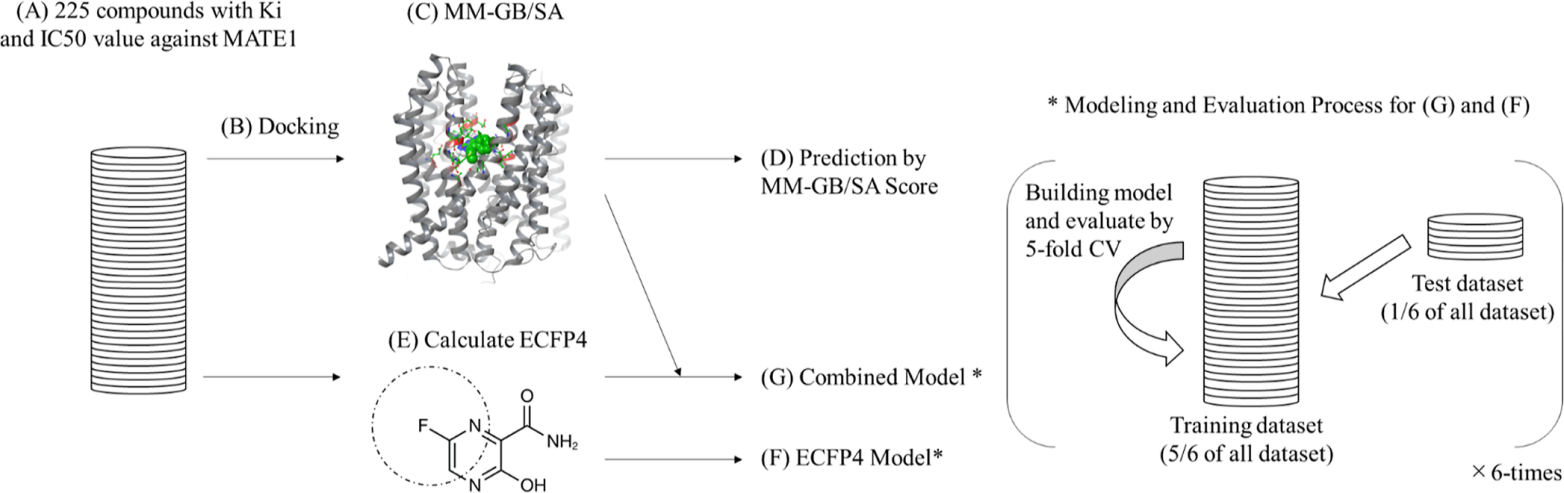
Overview of the workflow of this research. (A) The compounds were collected from DIDB database. (B) The compounds were docked into the MATE1 structure obtained from Alpha fold. (C) Using the docked pose MM–GB/SA calculation was performed. (D) The MM–GB/SA scores were used for the estimation of inhibitory activities. (E) The ECFP4 of the compounds were calculated. (F) A RF model against MATE1 inhibitory activity using ECFP4 was built. (G) Both MM–GB/SA scores and ECFP4 were used to construct another RF model against MATE1 inhibitory activity. * indicates the evaluation flow of RF models with six times five-fold CV.

The workflow in this study is shown in Figure 1. The compounds in the data set were docked, and the binding energies were calculated by the MM–GB/SA method. Then, the fingerprints were also calculated and used for the ML model. Additionally, an ML model which combines MM–GB/SA scores and fingerprints was built. The explanation of each step in detail was described below.

### 2.1 Data Set Collection

We collected *K*_*i*_ and IC50 data of MATE1 inhibitory activity from scientific literature and documents of New Drug Application for Food Drug Authority in the US via DIDB (Figure 1A).^29^ The total number of compounds was 225. The data are shown in Table S1.

### 2.2 Data Labeling for Classification of Inhibitory Activity against MATE1

For the MATE1 inhibitors that have showed clinically relevant DDI with metformin, most of inhibitors have the *K*_*i*_ value of less than 10 μmol/L for MATE1 such as cimetidine, pyrimethamine, trimethoprim, and vandetanib (isabuconazole and ranolazine have no data for *K*_*i*_ against metformin in this report).^30^ IC50 can be converted to *K*_*i*_ using the Cheng–Prusoff equation assuming the competitive inhibition^31^
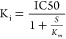
where *S* is the substrate concentration and *K*_m_ is the Michaelis–Menten constant of the substrate. Generally, in order to avoid the saturation of transportability of the substrate in the transporter, inhibition assays are conducted in the condition of *K*_m_ ≫ *S*, which shows *K*_*i*_ equals IC50. Therefore, collected IC50 were used assuming they equal *K*_*i*_. Therefore, in this study, 10 μmol/L was set as the reference criteria to judge whether the MATE1-mediated DDI risk can be negligible or not. In total, the number of compounds labeled as positive (inhibitor) is 101 and as negative (noninhibitor) is 124.

### 2.3 Standardization of Compound Representations

All simplified molecular-input line-entry system (SMILES) strings were obtained from PubChem manually.^32^ All structures were canonicalized using the RDKit (version 2020.09.01) components “RDKit Canon SMILES” and “Speedy SMILES desalt” in KNIME (version 4.3.4).^33^

### 2.4 Data Set Distribution in Chemical Space Compared to Approved Drugs Using ECFP4

We investigated the chemical space present in the data set comparing United States Food and Drug Administration (FDA)-approved drugs from DrugBank (version5.1.8)^34^ through using uniform manifold approximation and projection (UMAP).^35^ For the input, we calculated a similarity matrix with extended-connectivity fingerprints with radius 4 (ECFP4) (1024 bit, radius: 2) chemical descriptors.^36^ ECFP4 was calculated using the corresponding RDKit (version 2020.09.01) Chem functions^37^ AllChem.

### 2.5 MM–GB/SA

#### 2.5.1 Protein Preparation

No human MATE1 protein structures have so far been solved, and only closely related proteins represent a suitable template for homology modeling; however, the identity of human MATE1 to the related X-ray resolved proteins, NorM-Vc, PfMATE, and DTX14-At,^38^ was 23.60, 22.40, and 29.61%, respectively. Moreover, the MATE1 is a membrane protein that is known to have a difficulty to reproduce its movement by MD simulations because the membrane is composed of too many kinds of lipids to reproduce in silico^39^^,^.^40^ Consequently, the AlphaFold^41^ model of human MATE1 was used as a template of docking simulations. The model (AF-Q96FL8-F1-model_v4.pdb) was downloaded^42^ and processed for docking by assigning bond orders, adding hydrogen atoms and optimization of hydrogen atom positions, followed by optimization of hydrogen bond and whole complex energy minimization using the protein preparation wizard of Schrodinger Suite 2021–2.^43^

#### 2.5.2 Docking Study

Before docking simulation, sitemap was used for the identification of the binding site of MATE1 (Schrodinger Suite 2021–2).^40^ The site with the highest SiteScore was selected as the binding site (Figure S1). We used the Glide SP^16^, using the OPLS-2005^44^ force field and the following parameters: a protein van der Waals (vdW) radius scaling of 1.0, a ligand vdW radius scaling of 0.80, and a grid size (centered on the amino acid residues within 4 Å of the ligand in the cocrystallized X-ray structure) of 10 × 10 × 10 Å^3^ (Figure 1B). Regarding ligands, using the SMILES strings mentioned before, three-dimensional conformations were generated using LigPrep (pH 7 was set for protonation state calculation),^45^ followed by multiple conformer generation using Confgen.^46^ In cases of multiple possible stereoisomers, tautomer, and protonation states for a single ligand, we selected the one which corresponds to the minimum energy conformation. Docking was performed without specific interaction controls with individual amino acids in GLIDE,^16^ generating the one lowest energy docking pose per ligand. During docking, a maximum of 5000 docking poses was obtained at first, of which the best-scoring 400 poses were minimized and rescored. Finally, the best-scoring pose was retained for further analysis.

#### 2.5.3 Set Up for MM–GB/SA Calculation

We obtained a MM–GB/SA score, which measures the binding free energy (*G*_bind_) in the ligand–protein complex at single point using the MMGB/SA method in the Prime program (Prime MMGBSA v3.000).^47^ The docked poses were minimized around amino acids within 3 Å from the inhibitors using the local optimization feature in Prime, and the energies of the complexes were calculated using the OPLS4 force field and GB/SA continuum solvent model (Figure 1C). The *G*_bind_ was then estimated using the equation
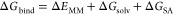
where Δ*E*_MM_ was the difference in energy between the ligand–protein complex and the sum of the energies of the ligand and free protein, using the OPLS-2005 force field; Δ*G*_solv_ was the difference in the GB/SA solvation energy between the ligand–protein complex and the sum of the solvation energies for the ligand and free protein; and Δ*G*_SA_ was the difference in the surface area energy between the ligand–protein complex and the sum of the surface area energies for the ligand and free protein. Corrections for entropic changes were not applied. The dielectric constants were set according to the solvent water (VSGB2.1 solvation model).

#### 2.5.4 Linear Regression Model Using Glide Docking Score and MM–GB/SA Score and the Metrics for the Evaluation

To compare the model validity between GLIDE docking and MM–GB/SA score against the pIC50 value of MATE1 inhibitory activity, we performed a linear regression (Figure 1D) and calculated *R*^2^-values. The equation was as follows where SS_res_ means sum of squared of residuals and SS_tot_ means total sum of squares. This calculation was performed in Microsoft Excel (version Office 365) manually.
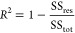


### 2.6 Machine Learning

#### 2.6.1 Data Split

The data set was randomly divided into training (5/6 of all) and test data sets (1/6 of all). For machine learning, this selection process of training and test data set was repeated six times to perform hold-out tests six times without replacement in test data set for different runs. The hold-out compound ID in each run is included in Table S1.

#### 2.6.2 Machine Learning Models and Their Explanatory Variables

For machine learning modeling, chemical descriptors ECFP4 (Figure 1E) and compound SMILES themselves were used for RF and message passing neural network (MPNN), respectively. We first investigated the machine learning models (RF and MPNN) using only ECFP4 fingerprints (Figure 1F). Then, in order to investigate the effect of parallel usage of different types of variables as explanatory ones, we built the models with both ECFP4 and MM–GB/SA scores (Figure 1G). Regarding RF, the Python (ver. 3.7.10) scikit-learn (version 0.24.2) library RandomForest Regressor function was used, and the parameters were set as defaults.^48^ Regarding MPNN, the Python (ver. 3.7.10) Chemprop (version 1.3.1) library chemprop function was used, and the parameters were set as defaults.^49,50^

### 2.7 Model Validation

Five-fold CV (five-fold) was performed using the KFold function in scikit-learn (versions 0.24.2) with parameters: n_splits = 5 and shuffle = True. Furthermore, hold-out test was performed with a separate test data set. To avoid the biased data set composition, we repeated these processes six times (Figure 1F,G).

### 2.8 Model Metrics

In the five-fold CV, AUC-ROC and accuracy were calculated to quantify the model performance. In the hold-out test, in addition to AUC-ROC and accuracy, sensitivity, specificity, Youden’s index, Matthews correlation coefficient, false rate, and F-measure were calculated using the confusion matrixes in Microsoft Excel (version Office 365) in order to examine the extrapolation of the model in detail.

### 2.9 Estimation of the Contribution of MM–GB/SA Scores in the Predictive RF Model

To investigate the contribution of MM–GB/SA scores in the predictive RF model (and to ensure that dimensionality of features employed was not a problem), all of the data set was used to construct a full model, and Gini importance of MM–GB/SA scores against the others was calculated. The Python (ver. 3.7.10) scikit-learn (version 0.24.2) library RandomForest Regressor function and feature_importances function were used; the total value of Gini importance was set to be 1.0.

### 2.10 5-Nearest Neighbor Analysis to Investigate the Applicability Domain for Each Model

The distribution of Tanimoto similarity of 5-nearest neighbors (NN) was calculated by ECFP4 using the RDKit (version 2020.09.01)^37^ Chem functions. We set each bin according to the Tanimoto similarity as less than 0.2, 0.2 to 0.3, 0.3 to 0.4, and more than 0.4 (since lower and larger thresholds were not populated with sufficient numbers of data points) and plotted the percentage of compounds predicted correctly and incorrectly against these bins to investigate whether we can see the applicability domain through the predictivity of models built in this study.

### 2.11 Structural Interaction Fingerprints for the Analysis of Interaction Amino Acids

To estimate the important amino acids for the inhibition of MATE1 activity, we calculated the structural interaction fingerprints (SIFt)^51,52^ between the ligands and MATE1 using the interaction fingerprints in Maestro (Schrodinger Suite 2021–2).^40^ The interaction patterns between the protein and the inhibits and noninhibitors were compared. We drew a Venn-diagram for the number of interacted amino acids against inhibitors and noninhibitors using the matplotlib_venn (versions 0.11.10) venn2 function. We picked the amino acids that interacted with inhibitors more than 10 times, and then we calculated the ratio of the number of interactions with amino acids among inhibitors vs noninhibitors.

## 3 Results and Discussion

### 3.1 Analysis of Chemical Space Comparing FDA Compounds

First, to investigate the chemical space of compounds collected in this study, UMAP analysis was performed by comparing our compounds to FDA approved drugs. The result of this analysis is shown in Figure S2 where it can be seen that the chemical space of compounds used in this study for predictive model building were consistent with that of this compound set. It should be kept in mind that visualizations can easily be misleading; hence, the results section contains a more detailed applicability domain analysis in this regard.

### 3.2 Validation of Docking Poses for Cimetidine

Next, in order to examine the validity of the docking pose used in this study, we checked the docking pose of cimetidine. Because this compound is a clinical MATE inhibitor,^10,12^ although no bound X-ray structure is published, we can compare the docking poses obtained in our work to a previous study^53^^,^. The results are shown in Figure S3A,B. The NH moiety of the azole formed a hydrogen bond with Glu389, the azole ring can also form a π-interaction with Tyr299, and the cyanoguanidine moiety formed a hydrogen bond with Gln49. The interactions with Glu389 and Tyr299 were consistent with the previous report; and although the interaction with Gln49 was not reported, the position of the compound was consistent with the previous study.^50^ Hence, we concluded that the quality of our docking poses passed this plausibility check by comparison to a previous study.

### 3.3 Correlation between Inhibitory Activity in MATE1 Transporter and MM–GB/SA Score

**Figure 2:**
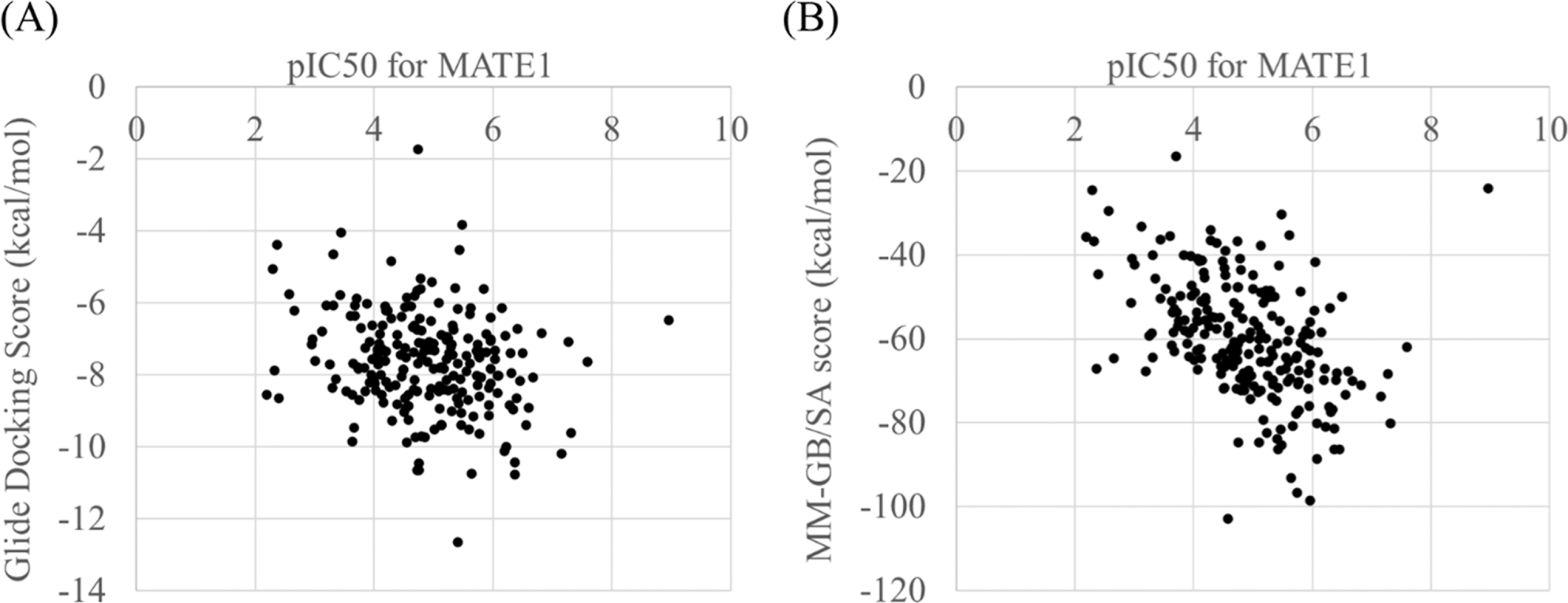
Correlation of docking score and MM–GB/SA score against pIC50 values for MATE1 transporters.

Next, we compared docking and MM–GB/SA scores to pIC50-values against the MATE1 transporter, which is shown in Figure 2A,B, respectively. Based on both visual inspection and *R*^2^ value, the MM–GB/SA score (0.196) is a better indicator than the docking score (0.051) for MATE1 pIC50, albeit both values are rather low in absolute terms. When we remove an outlier from the MM–GB/SA score plot (gabapentin: pIC50 8.96 and MM–GB/SA score: −24.19), the *R*^2^ value became much better (0.262). This is consistent with many results from previous research which claims that MM–GB/SA can predict the binding affinity of ligand–protein better than docking because of considering solvation energy and entropy.^22−26^ Hence, we can conclude that the MM–GB/SA score is a promising candidate for the prediction of the inhibitory activity against MATE1.

We can see a better correlation of scores against pIC50 in MM–GB/SA (B) than that in docking scores (A).

### 3.4 Prediction of Inhibitory Activity by MM–GB/SA Score, RF (ECFP4) Model, MPNN (Graph) Model of MATE1 Transporter, and Combinations Thereof

**Figure 3:**
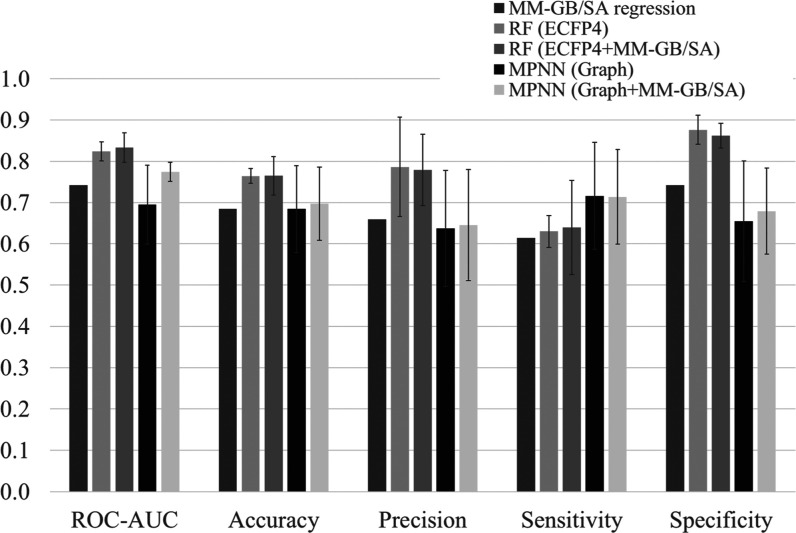
Metrics of classification models in the hold-out test set compared to the MM–GB/SA regression model.

We next built machine learning classification models for MATE1 inhibition at a 10 μmol/L cutoff threshold, based on ECFP4 fingerprints with the RF model, and graph input for the MPNN model. Main metrics for the hold-out test are shown in Figure 3, and the detailed values including other metrics are shown in Table S2. Regarding the MM–GB/SA regression model (Figure 2B), if the predicted value is below 10 μmol/L, the predicted class is considered positive. Conversely, if the predicted value exceeds 10 μmol/L, the predicted class is considered negative. When applying the classification threshold, overall accuracy and ROC-AUC were relatively high at 0.684 and 0.742, respectively, and its specificity was 0.742; precision and sensitivity were somewhat lower at 0.660 and 0.614, respectively. However, considering that the best AUC of the previous best ML model was 0.78 [*J. Med. Chem.***2013**, *56*(3), 781–795],^14^ we can see that MM–GB/SA scores have a large impact on the prediction of MATE1 inhibitory activity.

Regarding the RF model using ECFP4 fingerprints, the ROC-AUC and averaged accuracy were very high (0.824 and 0.765, respectively), and comparing those in five-fold CV (0.810 and 0.741), the overfitting was considered to be avoided. The precision and specificity were also high (0.786 and 0.876, respectively). However, since the sensitivity was relatively low (0.630), the concern with this model was to miss potential MATE1 inhibitors.

The RF model using both ECFP4 and MM–GB/SA score as input features resembles in its metrics the RF model using only ECFP4 fingerprints. Specifically, this model had high values for ROC-AUC (0.833), averaged accuracy (0.765), precision (0.779), specificity (0.862), and a relatively low value for sensitivity (0.640). Nevertheless, the ROC-AUC was 0.833, which is both a better value than that reported in previous studies and the best value obtained here. The ROC-AUC and averaged accuracy of five-fold CV of this model were 0.823 and 0.741, respectively, which were similar to those in the hold-out test set, and hence, this model had little concern regarding overfitting. Furthermore, we investigated the Gini importance of MM–GB/SA in this model and found that the MM–GB/SA score had the highest impact in the explanatory variables (0.053 out of 1.0 in total); the second and third ones from ECFP4 were 0.036 and 0.016. Consequently, we conclude that the MM–GB/SA score was identified as being important in this model, despite the much higher dimensionality of the ECFP4 feature set.

Regarding the conventional MPNN model, the ROC-AUC was 0.695, which was lower than the MM–GB/SA regression model, and the accuracy was 0.684, which was identical to it. Respective values from five-fold CV were 0.710 and 0.641, which indicate no significant overfitting. The precision and specificity were lowest in this study (at 0.637 and 0.655, respectively). However, the sensitivity was higher (0.716) than those of other models (0.614–0.640). Consequently, we can see that this MPNN model has lower predictivity in overall metrics; however, its high sensitivity is beneficial to not miss any inhibitors, which is a very important aspect in practical project work.

Regarding the MPNN model using MM–GB/SA scores, by comparing the ROC-AUC of the conventional MPNN, an improvement was seen from 0.695 to 0.774, while those related to averaged accuracy (0.698), precision (0.645), and specificity (0.679) were marginal (0.01–0.02). The sensitivity was mostly the same as that of the conventional MPNN, at a relatively high value of 0.714. Additionally, since the ROC-AUC and averaged accuracy of five-fold CV were 0.769 and 0.681, respectively, which were similar to those in hold-out test, this model has little concern about over fitting. Consequently, we can see that the benefit of the MM–GB/SA score for the overall predictivity in the MPNN model, and also its high sensitivity, can compensate for the defects of MM–GB/SA regression models and RF models.

Error bars were calculated based on the results of the sixth repeated hold-out test. The best ROC-AUC and accuracy were observed in the RF model, and the positive effect of the MM–GB/SA score was observed in MPNN clearly.

### 3.5 5-NN Analysis

**Figure 4:**
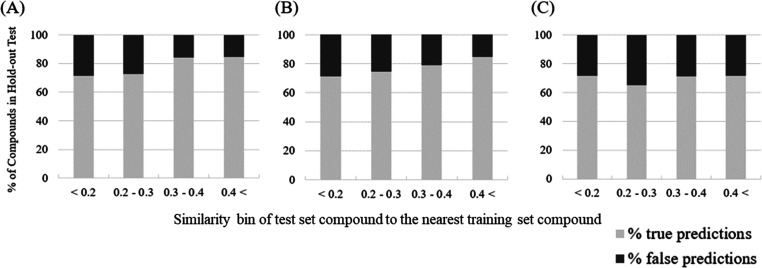
5-NN analysis using Tanimoto similarity of ECFP4 of test set compounds to nearest neighbor from the training set. (A) RF model using only ECFP4, (B) RF model using both ECFP4 and MM–GB/SA, and (C) MM–GB/SA regression model. It can be seen that the data-driven model (i.e., ML model) showed a relatively strong dependency of prediction accuracy on similarity to the training set, while the physics-based model was virtually independent of it.

We next investigated the applicability domain of two RF models, based on only ECFP4 fingerprints and based on both ECFP4 and MM–GB/SA scores, by 5-NN analysis using Tanimoto similarity of ECFP4 fingerprints. According to one practical implementation, if more compounds are predicted correctly in a higher Tanimoto similarity bin (TSB) in a 5-NN analysis, we can say this model has an interpretable applicability domain, which we can use to estimate prediction confidence for a new compound in a given 5-NN similarity bin.^54,55^ The results of this analysis are shown in Figure 4A where it can be seen that in the RF model using only ECFP4, the percentage of correctly predicted compounds increases according to the Tanimoto similarity, from 71% in the 0.1 to 0.2 of TSB, over 73% (0.2–0.3 of TSB) and 84% (0.3–0.4 of TSB), to 85% (more than 0.4 of TSB). The second results of this analysis are shown in Figure 4B where it can be seen that in the RF model using both ECFP4 and MM–GB/SA score, the percentage of correctly predicted compounds also increases according to the Tanimoto similarity, from 71% (0.1–0.2 of TSB), over 75% (0.2–0.3 of TSB) and 79% (0.3–0.4 of TSB), to 85% (more than 0.4 of TSB). Therefore, based on these results, we can conclude that for the RF model, regardless of the explanatory variables, we see a correlation of the model performance with proximity to the training set. Finally, the result of the MM–GB/SA regression model is shown in Figure 4C where it can be seen that the percentage of correctly predicted compounds was largely independent of the Tanimoto similarity, ranging from 71% (0.1–0.2 of TSB), over 65% (0.2–0.3 of TSB), and 71% (0.3–0.4 of TSB) to 72% (more than 0.4 of TSB). Hence, the performance of the MM–GB/SA regression model is independent of similarity to the training set, which is due to MM–GB/SA based on the physics-based (instead of data-driven) model.^27,28^ From the practical viewpoint, likely parallel use of both models would be advised, to cover both the value of existing data and to take advantage of the data-agonist nature of MM–GB/SA scoring.

### 3.6 Structural Analysis of Binding Patterns via SIFts

**Figure 5:**
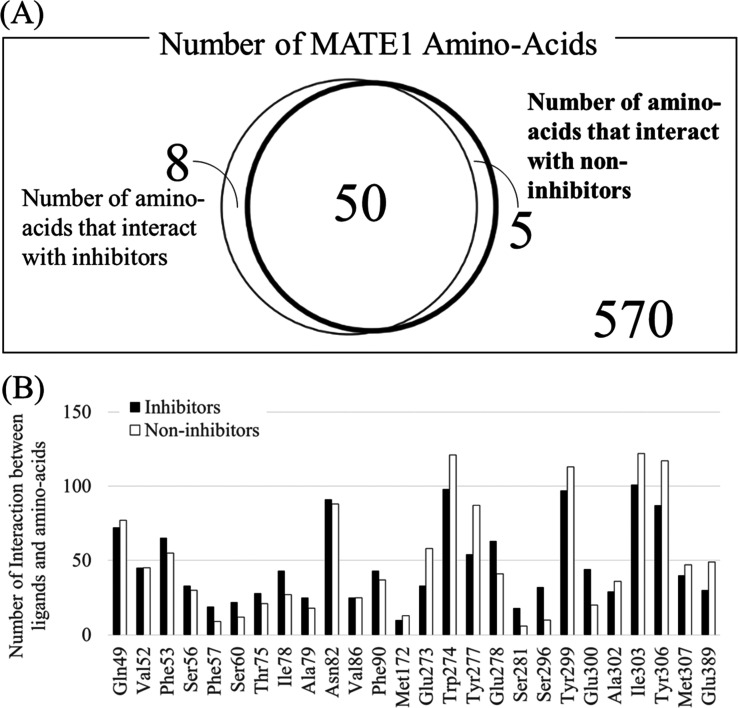
SIFts analysis (A) number of amino acids that interact at least once with inhibitors (thin circle) and noninhibitors (thick circle). (B) Amino acids that interact with inhibitors more than 10 times and interaction frequencies. It can be seen that the amino acids that interact with inhibitors and noninhibitors largely overlap, and they are hence insufficient to distinguish between both groups of compounds.

Next, in order to investigate the important amino acids, we estimated the amino acids of the MATE1 transporter which interact with inhibitors through SIFts. The result of this analysis is shown in Figure 5 where it can be seen that several amino acids emerged frequently as contacted amino acids, out of a total set of 570 amino acids present. The number of amino acids that interact with inhibitors was 58, and those with noninhibitors was 55. Interestingly, 50 amino acids were overlapped (Figure 5A). When we focused on amino acids that interact with more than 10 inhibitors, we found that Gln49, Ph53, Asn82, Trp274, Tyr277, Glu278, Tyr299, Ile303, and Tyr306 are involved in forming interactions (Figure 5B). Most of these amino acids are related to the contact with noninhibitors. Although only Glu278 had the larger number of contact in inhibitors than noninhibitors, the difference of it was not so large (63 inhibitors and 41 noninhibitors). Hence, we can conclude that the path of MATE1 for the inhibitors and noninhibitors are mostly the same.

In order to investigate the interactions of compounds with MATE1 further, we scrutinized the noninhibitor compounds which are known as in vitro typical substrates in FDA guidance,^10^ 1-methyl-4-phenylpyridinium, tetraethylammonium, creatinine, and metformin. The contacted amino acids with these substrates were Gln49, Glu273, Trp274, Tyr277, Tyr299, Ala302, Ile303, Tyr306, Met307, and Glu389. Since most of these amino acids (Gln49, Trp274, Tyr277, Tyr299, Ile303, and Tyr306) were the same with the inhibitor contacting amino acids, we can conclude that substrate and inhibitor overall share rather similar binding space in MATE1.

To examine this similarity of amino acid contacting in both inhibitor and noninhibitor further, we checked the binding pose after MM–GB/SA in several compounds that were randomly picked up considering pIC50 range [gabapentin (pIC50:8.96), nemiralisib (pIC50:7.59), nitidine (pIC50:6.82), epinastine (pIC50:5.96), copanlisib (pIC50:4.97), methamphetamine (pIC50:3.97), and caffeine (pIC50:2.96)]. Figure S4A–G shows the docking poses where it can be seen that regardless of inhibitor or noninhibitor, the specified amino acids (Gln49, Trp274, Tyr277, Tyr299, Ile303, and Tyr306) were frequently interacting with the ligands. From the viewpoint of drug discovery using in silico technology, this indicates the difficulty to distinguish between classes based on the docking position, and this leads to the low predictivity of the MM–GBSA score. On the other hand, from the viewpoint of drug discovery using experimental science, this is consistent with the fact that the IC50 values of each inhibitor when using different substrates are similar;^5,56^ and practically this can release the experimental scientists from bothering of selecting substrates for MATE1.

## 4 Conclusions

In this work, we aimed to integrate ligand-based and structure-based information to arrive at a MATE1 inhibition model which can be used for IC50 prediction. We found that the overall best predictivity was observed in the RF model (including ECFP4 and MM–GB/SA input variables) with an ROC-AUC of 0.833. On the one hand, RF models have good precision and specificity; on the other hand, MPNN models have good sensitivity. Additionally, fingerprint-based models performed better with increasing proximity to the training set, which was shown not to be the case for a MM–GB/SA regression model due to its physics-based nature. *Via* SIFt analysis, we identified that residues relevant for inhibitor are mostly the same with noninhibitors including substrates, such as Gln49, Trp274, Tyr277, Tyr299, Ile303, and Tyr306. This is consistent with the fact that the IC50 values of each inhibitor when using different substrates are similar, and practically this can release the experimental scientists from the bothering of selecting substrates for MATE1. Hence, we were in the current study able to build a fit-for-purpose classification model for MATE1 inhibitory activity based on dose–response data based on both ECFP4 fingerprints and the MM–GB/SA score for the first time, which will be useful in practice to identify and eliminate more compounds related to MATE1-based DDI in the early drug discovery stages.

## Data Availability

All of the data that support the findings of this study are available in the Supporting Information.

## Supporting Information Available

Information on compounds used in this study; all metrics calculated for each model; prediction of binding sites; chemical space analysis of the data set used in this study and FDA-approved compounds by UMAP; docking pose of representative drugs, cimetidine; and picked docking poses of inhibitors and noninhibitors (PDF)

## Abbreviations

RF: random forest
MPNN: message passing neural network
ECFP4: extended-connectivity fingerprints with radius 4
MATE: multidrug and toxin extrusion transporter
OCT: organic cation transporter
DDI: drug–drug interactions
CV: cross validation
MM–GB/SA: molecular mechanics with generalized born and surface area solvation
SMILE: simplified molecular-input line-entry system
FDA: United States Food and Drug Administration
*G*_bind_: binding free energy
NN: nearest neighbors
SIFt: structural interaction fingerprints
TSBT: Tanimoto similarity bin
