# Supplementary material for: Prediction of Inhibitory Activity against the MATE1 Transporter via Combined Fingerprint- and Physics-Based Machine Learning Models
Source: J Chem Inf Model. 2024 Sep 10;64(18):7068–76. doi: 10.1021/acs.jcim.4c00921 (PMC11423340; doi:10.1021/acs.jcim.4c00921)
Supplement: Supplementary file 1 — ci4c00921_si_001.pdf [file ci4c00921_si_001.pdf]

Supporting Information

Prediction of Inhibitory Activity Against the MATE1 Transporter *via* Combined Fingerprint- and Physics-Based Machine Learning Models

Koichi Handa[1,2], Shunta Sasaki[3], Satoshi Asano[2], Michiharu Kageyama[2], Takeshi Iijima[2], Andreas Bender[1,4*]*

[1]Centre for Molecular Informatics, Department of Chemistry, University of Cambridge, Lensfield Road, Cambridge, CB2 1EW, UK.

[2]Toxicology & DMPK Research Department, Teijin Institute for Bio-medical Research, Teijin Pharma Limited, 4-3-2 Asahigaoka, Hino-shi, Tokyo 191-8512, Japan.

[3]Pharmaceutical Discovery Research Laboratories, Teijin Pharma Limited, Tokyo, Japan.

[4]Institutul STAR-UBB, Universitatea Babeș-Bolyai, Str. Mihail Kogălniceanu nr. 1, 400084, Cluj-Napoca, Romania.

Table S1 The summary of information of compounds used in this study

Data file is not shown here; it will be uploaded as an excel file.

ID	Name	Canonical Smiles	pIC50	Label	Run1	Run2	Run3	Run4	Run5	Run6
1	gabapentin	NCC1(CC(=O)O)CCCCC1	8.96	Positive	Train	Train	Hold-out test	Train	Train	Train
2	nemiralisib	CC(C)N1CCN(Cc2cnc(-c3cc(-c4cccc5[nH]ccc45)cc4[nH]ncc34)o2)CC1	7.59	Positive	Hold-out test	Train	Train	Train	Train	Train
3	N-desmethylimatinib CGP74588	Cc1ccc(NC(=O)c2ccc(CN3CCNCC3)cc2)cc1Nc1nccc(-c2cccnc2)n1	7.32	Positive	Train	Train	Train	Hold-out test	Train	Train
4	gilteritinib	CCc1nc(C(N)=O)c(Nc2ccc(N3CCC(N4CCN(C)CC4)CC3)c(OC)c2)nc1NC1CCOCC1	7.27	Positive	Train	Train	Hold-out test	Train	Train	Train
5	ondansetron	Cc1nccn1CC1CCc2c(c3ccccc3n2C)C1=O	7.16	Positive	Train	Train	Hold-out test	Train	Train	Train
6	nitidine	COc1cc2c[n+](C)c3c4cc5c(cc4ccc3c2cc1OC)OCO5	6.82	Positive	Train	Train	Train	Hold-out test	Train	Train
7	tucatinib	Cc1cc(Nc2ncnc3ccc(NC4=NC(C)(C)CO4)cc23)ccc1Oc1ccn2ncnc2c1	6.67	Positive	Train	Hold-out test	Train	Train	Train	Train
8	rilpivirine	Cc1cc(/C=C/C#N)cc(C)c1Nc1ccnc(Nc2ccc(C#N)cc2)n1	6.60	Positive	Train	Train	Train	Hold-out test	Train	Train
9	fedratinib	Cc1cnc(Nc2ccc(OCCN3CCCC3)cc2)nc1Nc1cccc(S(=O)(=O)NC(C)(C)C)c1	6.45	Positive	Train	Train	Train	Hold-out test	Train	Train
10	IMATINIB	Cc1ccc(NC(=O)c2ccc(CN3CCN(C)CC3)cc2)cc1Nc1nccc(-c2cccnc2)n1	6.41	Positive	Train	Train	Train	Train	Hold-out test	Train
11	N-desmethylabemaciclib M2	Cc1nc2c(F)cc(-c3nc(Nc4ccc(CN5CCNCC5)cn4)ncc3F)cc2n1C(C)C	6.40	Positive	Train	Hold-out test	Train	Train	Train	Train
12	sunitinib	CCN(CC)CCNC(=O)c1c(C)[nH]c(/C=C2\C(=O)Nc3ccc(F)cc32)c1C	6.37	Positive	Train	Hold-out test	Train	Train	Train	Train
13	tafenoquine	COc1cc(C)c2c(Oc3cccc(C(F)(F)F)c3)c(OC)cc(NC(C)CCCN)c2n1	6.36	Positive	Train	Train	Train	Train	Hold-out test	Train
14	vandetanib	COc1cc2c(Nc3ccc(Br)cc3F)ncnc2cc1OCC1CCN(C)CC1	6.31	Positive	Hold-out test	Train	Train	Train	Train	Train
15	presatovir	Cc1cn2nc([C@@H]3CCCCN3C(=O)c3cc(Cl)ccc3NS(C)(=O)=O)cc2nc1N1CC[C@H](N)C1	6.30	Positive	Train	Train	Hold-out test	Train	Train	Train
16	abemaciclib	CCN1CCN(Cc2ccc(Nc3ncc(F)c(-c4cc(F)c5nc(C)n(C(C)C)c5c4)n3)nc2)CC1	6.28	Positive	Train	Hold-out test	Train	Train	Train	Train
17	capmatinib	CNC(=O)c1ccc(-c2cnc3ncc(Cc4ccc5ncccc5c4)n3n2)cc1F	6.55	Positive	Train	Train	Hold-out test	Train	Train	Train
18	GABEXATE	CCOC(=O)c1ccc(OC(=O)CCCCCN=C(N)N)cc1	6.21	Positive	Train	Train	Train	Train	Train	Hold-out test
19	rucaparib	CNCc1ccc(-c2[nH]c3cc(F)cc4c3c2CCNC4=O)cc1	6.20	Positive	Hold-out test	Train	Train	Train	Train	Train
20	isorhamnetin	COc1cc(-c2oc3cc(O)cc(O)c3c(=O)c2O)ccc1O	6.49	Positive	Hold-out test	Train	Train	Train	Train	Train
21	CHLORHEXIDINE	NC(=NCCCCCCN=C(N)N=C(N)Nc1ccc(Cl)cc1)N=C(N)Nc1ccc(Cl)cc1	6.15	Positive	Train	Train	Hold-out test	Train	Train	Train
22	crizotinib	C[C@@H](Oc1cc(-c2cnn(C3CCNCC3)c2)cnc1N)c1c(Cl)ccc(F)c1Cl	6.23	Positive	Train	Train	Train	Train	Train	Hold-out test
23	TACRINE	Nc1c2c(nc3ccccc13)CCCC2	6.09	Positive	Train	Train	Train	Train	Train	Hold-out test
24	brigatinib	COc1cc(N2CCC(N3CCN(C)CC3)CC2)ccc1Nc1ncc(Cl)c(Nc2ccccc2P(C)(C)=O)n1	6.08	Positive	Train	Hold-out test	Train	Train	Train	Train
25	hydroxyitraconazole	CC(O)C(C)n1ncn(-c2ccc(N3CCN(c4ccc(OCC5COC(Cn6cncn6)(c6ccc(Cl)cc6Cl)O5)cc4)CC3)cc2)c1=O	6.08	Positive	Train	Hold-out test	Train	Train	Train	Train

26	cinchonidine	C=C[C@H]1CN2CC[C@H]1C[C@H]2[C@H](O)c1ccnc2ccccc12	6.03	Positive	Train	Train	Train	Hold-out test	Train	Train
27	FAMOTIDINE	NC(N)=Nc1nc(CSCCC(N)=NS(N)(=O)=O)cs1	6.04	Positive	Hold-out test	Train	Train	Train	Train	Train
28	EPINASTINE	NC1=NCC2c3ccccc3Cc3ccccc3N12	5.96	Positive	Train	Train	Train	Train	Hold-out test	Train
29	ketoconazole	CC(=O)N1CCN(c2ccc(OC[C@@H]3CO[C@@](Cn4ccnc4)(c4ccc(Cl)cc4Cl)O3)cc2)CC1	5.96	Positive	Train	Train	Train	Train	Train	Hold-out test
30	keto-itraconazole	CC(=O)C(C)n1ncn(-c2ccc(N3CCN(c4ccc(OC[C@@H]5CO[C@@](Cn6cncn6)(c6ccc(Cl)cc6Cl)O5)cc4)CC3)cc2)c1=O	5.96	Positive	Train	Train	Hold-out test	Train	Train	Train
31	pemigatinib	CCN1C(=O)N(c2c(F)c(OC)cc(OC)c2F)Cc2cnc3[nH]c(CN4CCOCC4)cc3c21	5.96	Positive	Hold-out test	Train	Train	Train	Train	Train
32	dronedarone	CCCCc1oc2ccc(NS(C)(=O)=O)cc2c1C(=O)c1ccc(OCCCN(CCCC)CCCC)cc1	5.94	Positive	Train	Train	Train	Hold-out test	Train	Train
33	rimegepant	N[C@@H]1c2cccnc2[C@H](OC(=O)N2CCC(n3c(=O)[nH]c4ncccc43)CC2)CC[C@H]1c1cccc(F)c1F	5.93	Positive	Train	Train	Hold-out test	Train	Train	Train
34	DONEPEZIL	COc1cc2c(cc1OC)C(=O)C(CC1CCN(Cc3ccccc3)CC1)C2	5.93	Positive	Train	Train	Train	Train	Train	Hold-out test
35	topotecan	CC[C@@]1(O)C(=O)OCc2c1cc1n(c2=O)Cc2cc3c(CN(C)C)c(O)ccc3nc2-1	5.89	Positive	Train	Train	Train	Hold-out test	Train	Train
36	ZAFIRLUKAST	COc1cc(C(=O)NS(=O)(=O)c2ccccc2C)ccc1Cc1cn(C)c2ccc(NC(=O)OC3CCCC3)cc12	5.89	Positive	Train	Train	Train	Train	Hold-out test	Train
37	sorafenib	CNC(=O)c1cc(Oc2ccc(NC(=O)Nc3ccc(Cl)c(C(F)(F)F)c3)cc2)ccn1	5.84	Positive	Train	Train	Train	Train	Hold-out test	Train
38	prazosin;TERAZOSIN	COc1cc2nc(N3CCN(C(=O)c4ccco4)CC3)nc(N)c2cc1OC	5.80	Positive	Train	Hold-out test	Train	Train	Train	Train
39	risperidone	Cc1nc2n(c(=O)c1CCN1CCC(c3noc4cc(F)ccc34)CC1)CCCC2	5.80	Positive	Train	Train	Train	Train	Train	Hold-out test
40	BUSPIRONE	O=C1CC2(CCCC2)CC(=O)N1CCCCN1CCN(c2ncccn2)CC1	5.77	Positive	Train	Hold-out test	Train	Train	Train	Train
41	PENTAMIDINE	N=C(N)c1ccc(OCCCCCOc2ccc(C(=N)N)cc2)cc1	5.77	Positive	Hold-out test	Train	Train	Train	Train	Train
42	O-desmethylgefitinib	Oc1cc2ncnc(Nc3ccc(F)c(Cl)c3)c2cc1OCCCN1CCOCC1	5.76	Positive	Train	Train	Train	Train	Train	Hold-out test
43	MITOXANTRONE	O=C1c2c(O)ccc(O)c2C(=O)c2c(NCCNCCO)ccc(NCCNCCO)c21	5.74	Positive	Train	Train	Train	Hold-out test	Train	Train
44	brexpiprazole metabolite DM-3411	O=c1ccc2ccc(OCCCCN3CCN(c4cccc5c4C=CS5=O)CC3)cc2[nH]1	5.74	Positive	Train	Train	Hold-out test	Train	Train	Train
45	cinchonine	C=C[C@H]1CN2CCC1C[C@@H]2[C@@H](O)c1ccnc2ccccc12	5.73	Positive	Train	Train	Train	Hold-out test	Train	Train
46	gefitinib	COc1cc2ncnc(Nc3ccc(F)c(Cl)c3)c2cc1OCCCN1CCOCC1	5.73	Positive	Train	Train	Hold-out test	Train	Train	Train
47	quinine	C=C[C@H]1CN2CC[C@H]1C[C@H]2[C@H](O)c1ccnc2ccc(OC)cc12	5.72	Positive	Train	Train	Hold-out test	Train	Train	Train
48	pazopanib	Cc1ccc(Nc2nccc(N(C)c3ccc4c(C)n(C)nc4c3)n2)cc1S(N)(=O)=O	5.96	Positive	Train	Train	Train	Train	Train	Hold-out test
49	DOMPERIDONE	O=c1[nH]c2ccccc2n1CCCN1CCC(n2c(=O)[nH]c3cc(Cl)ccc32)CC1	5.64	Positive	Train	Hold-out test	Train	Train	Train	Train
50	cobicistat	CC(C)c1nc(CN(C)C(=O)N[C@@H](CCN2CCOCC2)C(=O)N[C@H](CC[C@H](Cc2ccccc2)NC(=O)OCc2cncs2)Cc2ccccc2)cs1	5.63	Positive	Train	Train	Train	Train	Hold-out test	Train
51	AMILORIDE	NC(N)=NC(=O)c1nc(Cl)c(N)nc1N	5.62	Positive	Train	Train	Train	Train	Train	Hold-out test
52	palonosetron	O=C1c2cccc3c2[C@H](CCC3)CN1[C@@H]1CN2CCC1CC2	5.61	Positive	Train	Train	Train	Hold-out test	Train	Train
53	amisulpride	CCN1CCCC1CNC(=O)c1cc(S(=O)(=O)CC)c(N)cc1OC	5.58	Positive	Hold-out test	Train	Train	Train	Train	Train

54	tazemetostat	CCN(c1cc(-c2ccc(CN3CCOCC3)cc2)cc(C(=O)NCc2c(C)cc(C)[nH]c2=O)c1C)C1CCOCC1	5.58	Positive	Train	Hold-out test	Train	Train	Train	Train
55	brexpiprazole	O=c1ccc2ccc(OCCCCN3CCN(c4cccc5sccc45)CC3)cc2[nH]1	5.55	Positive	Train	Train	Train	Train	Hold-out test	Train
56	dihydroergotamine	CN1C[C@H](C(=O)N[C@]2(C)O[C@@]3(O)[C@@H]4CCCN4C(=O)[C@H](Cc4ccccc4)N3C2=O)C[C@@H]2c3cccc4[nH]cc(c34)C[C@H]21	5.55	Positive	Train	Train	Train	Hold-out test	Train	Train
57	AMIODARONE	CCCCc1oc2ccccc2c1C(=O)c1cc(I)c(OCCN(CC)CC)c(I)c1	5.48	Positive	Train	Train	Train	Hold-out test	Train	Train
58	nilotinib	Cc1cn(-c2cc(NC(=O)c3ccc(C)c(Nc4nccc(-c5cccnc5)n4)c3)cc(C(F)(F)F)c2)cn1	5.47	Positive	Train	Train	Hold-out test	Train	Train	Train
59	ribociclib	CN(C)C(=O)c1cc2cnc(Nc3ccc(N4CCNCC4)cn3)nc2n1C1CCCC1	5.77	Positive	Train	Train	Train	Train	Hold-out test	Train
60	GUANFACINE	NC(N)=NC(=O)Cc1c(Cl)cccc1Cl	5.46	Positive	Train	Train	Train	Train	Hold-out test	Train
61	chloroquine	CCN(CC)CCCC(C)Nc1ccnc2cc(Cl)ccc12	5.67	Positive	Train	Train	Train	Hold-out test	Train	Train
62	lorlatinib	C[C@H]1Oc2cc(cnc2N)-c2c(nn(C)c2C#N)CN(C)C(=O)c2ccc(F)cc21	5.43	Positive	Train	Train	Train	Hold-out test	Train	Train
63	erlotinib metabolite OSI-420	C#Cc1cccc(Nc2ncnc3cc(OCCOC)c(OCCO)cc23)c1.Cl	5.42	Positive	Hold-out test	Train	Train	Train	Train	Train
64	irinotecan	CCc1c2c(nc3ccc(OC(=O)N4CCC(N5CCCCC5)CC4)cc13)-c1cc3c(c(=O)n1C2)COC(=O)[C@]3(O)CC.Cl	5.42	Positive	Train	Train	Hold-out test	Train	Train	Train
65	ritonavir	CC(C)c1nc(CN(C)C(=O)N[C@H](C(=O)N[C@@H](Cc2ccccc2)C[C@H](O)[C@H](Cc2ccccc2)NC(=O)OCc2cncs2)C(C)C)cs1	5.41	Positive	Train	Train	Hold-out test	Train	Train	Train
66	CIMETIDINE	CN=C(NC#N)NCCSCc1nc[nH]c1C	5.48	Positive	Train	Train	Hold-out test	Train	Train	Train
67	indinavir	CC(C)(C)NC(=O)[C@@H]1CN(Cc2cccnc2)CCN1C[C@@H](O)C[C@@H](Cc1ccccc1)C(=O)N[C@H]1c2ccccc2C[C@H]1O	5.47	Positive	Train	Train	Hold-out test	Train	Train	Train
68	dasatinib	Cc1nc(Nc2ncc(C(=O)Nc3c(C)cccc3Cl)s2)cc(N2CCN(CCO)CC2)n1	5.40	Positive	Train	Train	Hold-out test	Train	Train	Train
69	prucalopride	COCCCN1CCC(NC(=O)c2cc(Cl)c(N)c3c2OCC3)CC1	5.40	Positive	Train	Hold-out test	Train	Train	Train	Train
70	proguanil	CC(C)N=C(N)N=C(N)Nc1ccc(Cl)cc1	5.36	Positive	Train	Hold-out test	Train	Train	Train	Train
71	RABEPRAZOLE	COCCCOc1ccnc(CS(=O)c2nc3ccccc3[nH]2)c1C	5.34	Positive	Train	Hold-out test	Train	Train	Train	Train
72	osimertinib	C=CC(=O)Nc1cc(Nc2nccc(-c3cn(C)c4ccccc34)n2)c(OC)cc1N(C)CCN(C)C	5.33	Positive	Train	Train	Hold-out test	Train	Train	Train
73	PHENTOLAMINE	Cc1ccc(N(CC2=NCCN2)c2cccc(O)c2)cc1	5.33	Positive	Train	Train	Hold-out test	Train	Train	Train
74	tazemetostat N-demethylated metabolite EPZ-6930 M5	Cc1cc(C)c(CNC(=O)c2cc(-c3ccc(CN4CCOCC4)cc3)cc(NC3CCOCC3)c2C)c(=O)[nH]1	5.32	Positive	Hold-out test	Train	Train	Train	Train	Train
75	pyrimethamine	CCc1nc(N)nc(N)c1-c1ccc(Cl)cc1	5.31	Positive	Train	Hold-out test	Train	Train	Train	Train
76	glasdegib	CN1CC[C@@H](NC(=O)Nc2ccc(C#N)cc2)C[C@@H]1c1nc2ccccc2[nH]1	5.31	Positive	Train	Train	Train	Train	Train	Hold-out test
77	granisetron	CN1[C@H]2CCC[C@@H]1CC(NC(=O)c1nn(C)c3ccccc13)C2	5.30	Positive	Train	Train	Train	Train	Train	Hold-out test
78	dolutegravir	C[C@@H]1CCO[C@H]2Cn3cc(C(=O)NCc4ccc(F)cc4F)c(=O)c(O)c3C(=O)N21	5.29	Positive	Train	Hold-out test	Train	Train	Train	Train
79	DX-619	COc1c(N2CC[C@@H](C3(N)CC3)C2)ccc2c(=O)c(C(=O)O)cn([C@@H]3C[C@@H]3F)c12	5.59	Positive	Train	Train	Hold-out test	Train	Train	Train
80	olaparib	O=C(c1cc(Cc2n[nH]c(=O)c3ccccc23)ccc1F)N1CCN(C(=O)C2CC2)CC1	5.26	Positive	Train	Hold-out test	Train	Train	Train	Train

81	osilodrostat	N#Cc1ccc([C@H]2CCc3cncn32)c(F)c1	5.25	Positive	Hold-out test	Train	Train	Train	Train	Train
82	cabozantinib	COc1cc2nccc(Oc3ccc(NC(=O)C4(C(=O)Nc5ccc(F)cc5)CC4)cc3)c2cc1OC	5.23	Positive	Train	Train	Train	Hold-out test	Train	Train
83	imperatorin	CC(C)=CCOc1c2occc2cc2ccc(=O)oc12	5.22	Positive	Train	Train	Train	Train	Hold-out test	Train
84	isavuconazole	C[C@@H](c1nc(-c2ccc(C#N)cc2)cs1)[C@](O)(Cn1cncn1)c1cc(F)ccc1F	5.22	Positive	Train	Train	Train	Hold-out test	Train	Train
85	PHENFORMIN	NC(N)=NC(N)=NCCc1ccccc1	5.21	Positive	Train	Train	Train	Train	Train	Hold-out test
86	flecainide	O=C(NCC1CCCCN1)c1cc(OCC(F)(F)F)ccc1OCC(F)(F)F	5.17	Positive	Train	Train	Train	Train	Hold-out test	Train
87	SUMATRIPTAN	CNS(=O)(=O)Cc1ccc2[nH]cc(CCN(C)C)c2c1	5.17	Positive	Train	Train	Train	Train	Train	Hold-out test
88	trimethoprim	COc1cc(Cc2cnc(N)nc2N)cc(OC)c1OC	5.25	Positive	Train	Train	Train	Hold-out test	Train	Train
89	RIMANTADINE	CC(N)C12CC3CC(CC(C3)C1)C2	5.14	Positive	Train	Train	Train	Hold-out test	Train	Train
90	lemborexant	Cc1ncc(OC[C@@]2(c3cccc(F)c3)C[C@H]2C(=O)Nc2ccc(F)cn2)c(C)n1	5.13	Positive	Train	Train	Train	Train	Train	Hold-out test
91	lemborexant metabolite M10	Cc1ncc(OC[C@@]2(c3cccc(F)c3)C[C@H]2C(=O)Nc2ccc(F)cn2)c(C)[n+]1[O-]	5.13	Positive	Train	Train	Train	Hold-out test	Train	Train
92	lemborexant metabolite M4	Cc1nc(C)[n+]([O-])cc1OC[C@@]1(c2cccc(F)c2)C[C@H]1C(=O)Nc1ccc(F)cn1	5.13	Positive	Train	Train	Train	Hold-out test	Train	Train
93	lemborexant metabolite M9	Cc1nc(CO)ncc1OC[C@@]1(c2cccc(F)c2)C[C@H]1C(=O)Nc1ccc(F)cn1	5.13	Positive	Train	Hold-out test	Train	Train	Train	Train
94	PROPRANOLOL	CC(C)NCC(O)COc1cccc2ccccc12	5.11	Positive	Train	Train	Train	Train	Train	Hold-out test
95	erlotinib	C#Cc1cccc(Nc2ncnc3cc(OCCOC)c(OCCOC)cc23)c1	5.10	Positive	Train	Hold-out test	Train	Train	Train	Train
96	bictegravir	O=C(NCc1c(F)cc(F)cc1F)c1cn2c(c(O)c1=O)C(=O)N1[C@H]3CC[C@H](C3)O[C@@H]1C2	5.10	Positive	Train	Train	Train	Train	Train	Hold-out test
97	dabigatran	Cn1c(CNc2ccc(C(=N)N)cc2)nc2cc(C(=O)N(CCC(=O)O)c3ccccn3)ccc21	5.09	Positive	Train	Train	Train	Train	Hold-out test	Train
98	butylscopolamine	CCCC[N+]1(C)[C@H]2CC(OC(=O)[C@H](CO)c3ccccc3)C[C@@H]1[C@H]1O[C@@H]21	5.09	Positive	Train	Hold-out test	Train	Train	Train	Train
99	esaxerenone	Cc1c(C(=O)Nc2ccc(S(C)(=O)=O)cc2)cn(CCO)c1-c1ccccc1C(F)(F)F	5.01	Positive	Train	Train	Train	Hold-out test	Train	Train
100	moxifloxacin	COc1c(N2C[C@@H]3CCCN[C@@H]3C2)c(F)cc2c(=O)c(C(=O)O)cn(C3CC3)c12	5.01	Positive	Train	Train	Hold-out test	Train	Train	Train
101	peficitinib	NC(=O)c1cnc2[nH]ccc2c1NC1[C@H]2CC3C[C@@H]1CC(O)(C3)C2	5.00	Negative	Train	Hold-out test	Train	Train	Train	Train
102	upadacitinib	CC[C@@H]1CN(C(=O)NCC(F)(F)F)C[C@@H]1c1cnc2cnc3[nH]ccc3n12	5.00	Negative	Train	Train	Train	Train	Hold-out test	Train
103	copanlisib	COc1c(OCCCN2CCOCC2)ccc2c3n(c(=NC(=O)c4cnc(N)nc4)nc12)CCN3	4.97	Negative	Hold-out test	Train	Train	Train	Train	Train
104	ranolazine	COc1ccccc1OCC(O)CN1CCN(CC(=O)Nc2c(C)cccc2C)CC1	4.95	Negative	Train	Train	Train	Train	Hold-out test	Train
105	baloxavir	O=C1c2c(O)c(=O)ccn2N([C@@H]2c3ccccc3SCc3c2ccc(F)c3F)[C@@H]2COCCN12	4.95	Negative	Hold-out test	Train	Train	Train	Train	Train
106	trospium	O=C(OC1C[C@@H]2CC[C@H](C1)[N+]21CCCC1)C(O)(c1ccccc1)c1ccccc1	4.94	Negative	Train	Train	Hold-out test	Train	Train	Train
107	atropine	CN1[C@H]2CC[C@@H]1CC(OC(=O)C(CO)c1ccccc1)C2	4.93	Negative	Train	Train	Hold-out test	Train	Train	Train
108	PROCHLORPERAZINE	CN1CCN(CCCN2c3ccccc3Sc3ccc(Cl)cc32)CC1	4.92	Negative	Train	Train	Hold-out test	Train	Train	Train
109	apalutamide	CNC(=O)c1ccc(N2C(=S)N(c3cnc(C#N)c(C(F)(F)F)c3)C(=O)C23CCC3)cc1F	4.86	Negative	Hold-out test	Train	Train	Train	Train	Train
110	imiquimod	CC(C)Cn1cnc2c(N)nc3ccccc3c21	4.86	Negative	Train	Hold-out test	Train	Train	Train	Train

111	temsavir metabolite BMS-646915	COc1cnc(-n2cnc(C)n2)c2[nH]cc(C(=O)C(=O)N3CCNCC3)c12	4.82	Negative	Train	Train	Train	Train	Train	Hold-out test
112	nuciferin	COc1cc2c3c(c1OC)-c1ccccc1C[C@H]3N(C)CC2	4.80	Negative	Train	Train	Train	Train	Hold-out test	Train
113	ESMOLOL	COC(=O)CCc1ccc(OCC(O)CNC(C)C)cc1	4.79	Negative	Train	Hold-out test	Train	Train	Train	Train
114	psoralen	O=c1ccc2cc3ccoc3cc2o1	4.79	Negative	Hold-out test	Train	Train	Train	Train	Train
115	PANTOPRAZOLE	COc1ccnc(CS(=O)c2nc3ccc(OC(F)F)cc3[nH]2)c1OC	4.79	Negative	Train	Train	Train	Train	Train	Hold-out test
116	cabozantinib 6-desmethyl amide cleavage product sulfate	COc1cc2nccc(Oc3ccc(NC(=O)C4(C(=O)O)CC4)cc3)c2cc1OS(=O)(=O)O	4.78	Negative	Train	Train	Train	Train	Hold-out test	Train
117	maraviroc	Cc1nnc(C(C)C)n1C1C[C@@H]2CC[C@H](C1)N2CC[C@H](NC(=O)C1CCC(F)(F)CC1)c1ccccc1	4.76	Negative	Train	Train	Train	Hold-out test	Train	Train
118	N-desmethylapalutamide	N#Cc1ncc(N2C(=O)C3(CCC3)N(c3ccc(C(N)=O)c(F)c3)C2=S)cc1C(F)(F)F	4.75	Negative	Train	Train	Train	Train	Train	Hold-out test
119	tramadol	COc1cccc([C@@]2(O)CCCC[C@@H]2CN(C)C)c1	4.75	Negative	Train	Train	Train	Train	Hold-out test	Train
120	TELMISARTAN	CCCc1nc2c(C)cc(-c3nc4ccccc4n3C)cc2n1Cc1ccc(-c2ccccc2C(=O)O)cc1	4.75	Negative	Train	Hold-out test	Train	Train	Train	Train
121	tetrabutylammonium	CCCC[N+](CCCC)(CCCC)CCCC	4.74	Negative	Train	Train	Train	Hold-out test	Train	Train
122	cabotegravir	C[C@H]1CO[C@@H]2Cn3cc(C(=O)NCc4ccc(F)cc4F)c(=O)c(O)c3C(=O)N12	4.74	Negative	Hold-out test	Train	Train	Train	Train	Train
123	temsavir	COc1cnc(-n2cnc(C)n2)c2[nH]cc(C(=O)C(=O)N3CCN(C(=O)c4ccccc4)CC3)c12	4.73	Negative	Train	Train	Train	Hold-out test	Train	Train
124	spironolactone	CC(=O)S[C@@H]1CC2=CC(=O)CC[C@]2(C)[C@H]2CC[C@@]3(C)[C@@H](CC[C@@]34CCC(=O)O4)[C@@H]21	4.73	Negative	Train	Train	Train	Hold-out test	Train	Train
125	homatropine	CN1[C@H]2CC[C@@H]1CC(OC(=O)C(O)c1ccccc1)C2	4.73	Negative	Train	Train	Train	Train	Hold-out test	Train
126	hydroxyabemaciclib M20	CCN1CCN(Cc2ccc(Nc3ncc(F)c(-c4cc(F)c5nc(CO)n(C(C)C)c5c4)n3)nc2)CC1	6.34	Positive	Train	Hold-out test	Train	Train	Train	Train
127	elagolix	COc1cccc(-c2c(C)n(Cc3c(F)cccc3C(F)(F)F)c(=O)n(C[C@H](NCCCC(=O)O)c3ccccc3)c2=O)c1F	4.72	Negative	Train	Train	Train	Train	Hold-out test	Train
128	OMEPRAZOLE	COc1ccc2nc(S(=O)Cc3ncc(C)c(OC)c3C)[nH]c2c1	4.72	Negative	Train	Train	Hold-out test	Train	Train	Train
129	quinidine	C=C[C@H]1CN2CCC1C[C@@H]2[C@@H](O)c1ccnc2ccc(OC)cc12	4.84	Negative	Hold-out test	Train	Train	Train	Train	Train
130	NORFLOXACIN	CCn1cc(C(=O)O)c(=O)c2cc(F)c(N3CCNCC3)cc21	4.70	Negative	Train	Train	Hold-out test	Train	Train	Train
131	CLONIDINE	Clc1cccc(Cl)c1NC1=NCCN1	4.68	Negative	Train	Train	Train	Train	Train	Hold-out test
132	(-)-tetrahydropalmatine	COc1cc2c(cc1OC)[C@@H]1Cc3ccc(OC)c(OC)c3CN1CC2	4.68	Negative	Hold-out test	Train	Train	Train	Train	Train
133	ethinylestradiol	C#C[C@]1(O)CC[C@H]2[C@@H]3CCc4cc(O)ccc4[C@H]3CC[C@@]21C	4.68	Negative	Hold-out test	Train	Train	Train	Train	Train
134	KETOTIFEN	CN1CCC(=C2c3ccccc3CC(=O)c3sccc32)CC1	4.67	Negative	Train	Train	Train	Train	Hold-out test	Train
135	selexipag	CC(C)N(CCCCOCC(=O)NS(C)(=O)=O)c1cnc(-c2ccccc2)c(-c2ccccc2)n1	4.66	Negative	Train	Train	Train	Hold-out test	Train	Train
136	ranitidine	CN/C(=C\[N+](=O)[O-])NCCSCc1ccc(CN(C)C)o1	4.67	Negative	Hold-out test	Train	Train	Train	Train	Train
137	naloxone	C=CCN1CC[C@]23c4c5ccc(O)c4O[C@H]2C(=O)CC[C@@]3(O)[C@H]1C5	4.62	Negative	Train	Train	Hold-out test	Train	Train	Train
138	diltiazem	COc1ccc([C@@H]2Sc3ccccc3N(CCN(C)C)C(=O)[C@@H]2OC(C)=O)cc1	4.90	Negative	Train	Train	Train	Train	Train	Hold-out test

139	DIPYRIDAMOLE	OCCN(CCO)c1nc(N2CCCCC2)c2nc(N(CCO)CCO)nc(N3CCCCC3)c2n1	4.59	Negative	Train	Train	Train	Hold-out test	Train	Train
140	ASTEMIZOLE	COc1ccc(CCN2CCC(Nc3nc4ccccc4n3Cc3ccc(F)cc3)CC2)cc1	4.58	Negative	Train	Train	Train	Hold-out test	Train	Train
141	kaempferol	O=c1c(O)c(-c2ccc(O)cc2)oc2cc(O)cc(O)c12	4.57	Negative	Train	Train	Hold-out test	Train	Train	Train
142	dasabuvir metabolite M1	COc1c(-c2ccc3cc(NS(C)(=O)=O)ccc3c2)cc(-n2ccc(=O)[nH]c2=O)cc1C(C)(C)CO	4.55	Negative	Train	Train	Train	Train	Train	Hold-out test
143	nizatidine	CN/C(=C\[N+](=O)[O-])NCCSCc1csc(CN(C)C)n1	4.54	Negative	Train	Train	Train	Train	Train	Hold-out test
144	serotonin	NCCc1c[nH]c2ccc(O)cc12	4.54	Negative	Hold-out test	Train	Train	Train	Train	Train
145	fosnetupitant	Cc1ccccc1-c1cc(N2CC[N+](C)(COP(=O)([O-])O)CC2)ncc1N(C)C(=O)C(C)(C)c1cc(C(F)(F)F)cc(C(F)(F)F)c1	4.53	Negative	Train	Train	Train	Train	Hold-out test	Train
146	selexipag metabolite ACT-333679	CC(C)N(CCCCOCC(=O)O)c1cnc(-c2ccccc2)c(-c2ccccc2)n1	4.52	Negative	Hold-out test	Train	Train	Train	Train	Train
147	ipratropium	CC(C)[N+]1(C)[C@H]2CC[C@@H]1CC(OC(=O)C(CO)c1ccccc1)C2	4.52	Negative	Train	Train	Train	Train	Train	Hold-out test
148	1-methyl-4-phenylpyridinium	C[n+]1ccc(-c2ccccc2)cc1	4.51	Negative	Train	Train	Train	Train	Hold-out test	Train
149	darolutamide	CC(O)c1cc(C(=O)N[C@@H](C)Cn2ccc(-c3ccc(C#N)c(Cl)c3)n2)n[nH]1	4.49	Negative	Train	Train	Train	Train	Hold-out test	Train
150	quercetin	O=c1c(O)c(-c2ccc(O)c(O)c2)oc2cc(O)cc(O)c12	4.48	Negative	Train	Train	Hold-out test	Train	Train	Train
151	noscapine	COc1ccc2c(c1OC)C(=O)O[C@@H]2[C@H]1c2c(cc3c(c2OC)OCO3)CCN1C	4.46	Negative	Train	Train	Train	Train	Train	Hold-out test
152	8-methoxypsoralen	COc1c2occc2cc2ccc(=O)oc12	4.45	Negative	Train	Train	Train	Train	Train	Hold-out test
153	temsavir metabolite BMS-930644	COc1cnc(-n2cnc(C)n2)c2[nH]cc(C(=O)C(=O)NCCNC(=O)c3ccccc3)c12	4.39	Negative	Train	Train	Train	Hold-out test	Train	Train
154	memantine	CC12CC3CC(C)(C1)CC(N)(C3)C2	4.39	Negative	Train	Train	Train	Hold-out test	Train	Train
155	fluperlapine	CN1CCN(C2=Nc3cc(F)ccc3Cc3ccccc32)CC1	4.39	Negative	Hold-out test	Train	Train	Train	Train	Train
156	(-)-epigallocatechin gallate	O=C(O[C@@H]1Cc2c(O)cc(O)cc2O[C@@H]1c1cc(O)c(O)c(O)c1)c1cc(O)c(O)c(O)c1	4.36	Negative	Train	Train	Train	Train	Train	Hold-out test
157	IMIPRAMINE	CN(C)CCCN1c2ccccc2CCc2ccccc21	4.53	Negative	Hold-out test	Train	Train	Train	Train	Train
158	VERAPAMIL	COc1ccc(CCN(C)CCCC(C#N)(c2ccc(OC)c(OC)c2)C(C)C)cc1OC	4.46	Negative	Hold-out test	Train	Train	Train	Train	Train
159	levofloxacin	C[C@H]1COc2c(N3CCN(C)CC3)c(F)cc3c(=O)c(C(=O)O)cn1c23	4.30	Negative	Train	Hold-out test	Train	Train	Train	Train
160	triethylmethylammonium	CC[N+](C)(CC)CC	4.29	Negative	Train	Train	Train	Train	Train	Hold-out test
161	para-hydroxymethamphetamine	CNC(C)Cc1ccc(O)cc1	4.23	Negative	Train	Hold-out test	Train	Train	Train	Train
162	varenicline	c1cnc2cc3c(cc2n1)C1CNCC3C1	4.21	Negative	Train	Hold-out test	Train	Train	Train	Train
163	tetrapropylammonium	CCC[N+](CCC)(CCC)CCC	4.20	Negative	Hold-out test	Train	Train	Train	Train	Train
164	cortisone	C[C@]12CC(=O)[C@H]3[C@@H](CCC4=CC(=O)CC[C@@]43C)[C@@H]1CC[C@]2(O)C(=O)CO	4.20	Negative	Train	Train	Train	Train	Hold-out test	Train
165	ORPHENADRINE	Cc1ccccc1C(OCCN(C)C)c1ccccc1	4.19	Negative	Train	Train	Train	Train	Hold-out test	Train
166	osilodrostat metabolite M34.5 LXB168	N#Cc1ccc(C2CCC3C(=O)NC(=O)C32)c(F)c1	4.17	Negative	Train	Train	Train	Train	Hold-out test	Train
167	veliparib	C[C@]1(c2nc3c(C(N)=O)cccc3[nH]2)CCCN1	4.16	Negative	Train	Train	Hold-out test	Train	Train	Train
168	3-hydroxycoumarin	O=c1oc2ccccc2cc1O	4.14	Negative	Hold-out test	Train	Train	Train	Train	Train

169	cortisol	C[C@]12C[C@H](O)[C@H]3[C@@H](CCC4=CC(=O)CC[C@@]43C)[C@@H]1CC[C@]2(O)C(=O)CO	4.13	Negative	Hold-out test	Train	Train	Train	Train	Train
170	baricitinib	CCS(=O)(=O)N1CC(CC#N)(n2cc(-c3ncnc4[nH]ccc34)cn2)C1	4.12	Negative	Train	Hold-out test	Train	Train	Train	Train
171	tetraethylammonium	CC[N+](CC)(CC)CC	4.09	Negative	Train	Train	Train	Train	Train	Hold-out test
172	SCOPOLAMINE	CN1C2CC(OC(=O)C(CO)c3ccccc3)CC1C1OC12	4.08	Negative	Train	Train	Train	Hold-out test	Train	Train
173	N,N,N-trimethyl-2-[methyl(7-nitrobenzo[c][l,2,5]oxadiazol-4-yl)amino]ethanaminium iodide	CN(CC[N+](C)(C)C)c1ccc([N+](=O)[O-])c2nonc12	4.07	Negative	Train	Train	Train	Train	Hold-out test	Train
174	tyramine	NCCc1ccc(O)cc1	4.06	Negative	Train	Train	Hold-out test	Train	Train	Train
175	pretomanid	O=[N+]([O-])c1cn2c(n1)OC[C@@H](OCc1ccc(OC(F)(F)F)cc1)C2	4.06	Negative	Train	Train	Hold-out test	Train	Train	Train
176	AMANTADINE	NC12CC3CC(CC(C3)C1)C2	4.28	Negative	Train	Hold-out test	Train	Train	Train	Train
177	amphetamine	CC(N)Cc1ccccc1	4.03	Negative	Train	Train	Train	Hold-out test	Train	Train
178	methamphetamine	CN[C@@H](C)Cc1ccccc1	3.97	Negative	Train	Train	Hold-out test	Train	Train	Train
179	MIDODRINE	COc1ccc(OC)c(C(O)CNC(=O)CN)c1	3.96	Negative	Train	Train	Train	Hold-out test	Train	Train
180	DESIPRAMINE	CNCCCN1c2ccccc2CCc2ccccc21	4.25	Negative	Hold-out test	Train	Train	Train	Train	Train
181	tryptoline	c1ccc2c3c([nH]c2c1)CNCC3	3.95	Negative	Train	Hold-out test	Train	Train	Train	Train
182	DISOPYRAMIDE	CC(C)N(CCC(C(N)=O)(c1ccccc1)c1ccccn1)C(C)C	4.13	Negative	Train	Train	Train	Hold-out test	Train	Train
183	CARVEDILOL	COc1ccccc1OCCNCC(O)COc1cccc2[nH]c3ccccc3c12	3.92	Negative	Train	Hold-out test	Train	Train	Train	Train
184	aripiprazole	O=C1CCc2ccc(OCCCCN3CCN(c4cccc(Cl)c4Cl)CC3)cc2N1	3.89	Negative	Train	Train	Hold-out test	Train	Train	Train
185	talipexole	C=CCN1CCc2nc(N)sc2CC1	4.18	Negative	Hold-out test	Train	Train	Train	Train	Train
186	ROLIPRAM	COc1ccc(C2CNC(=O)C2)cc1OC1CCCC1	3.85	Negative	Train	Train	Train	Train	Hold-out test	Train
187	nicotinamide mononucleotide	NC(=O)c1ccc[n+]([C@@H]2O[C@H](COP(=O)([O-])O)[C@@H](O)[C@H]2O)c1	3.83	Negative	Train	Train	Train	Train	Hold-out test	Train
188	nicotine	CN1CCC[C@H]1c1cccnc1	3.78	Negative	Train	Train	Train	Train	Train	Hold-out test
189	DIPHENHYDRAMINE	CN(C)CCOC(c1ccccc1)c1ccccc1	4.06	Negative	Train	Hold-out test	Train	Train	Train	Train
190	CHLORPHENIRAMINE	CN(C)CCC(c1ccc(Cl)cc1)c1ccccn1	4.06	Negative	Train	Train	Hold-out test	Train	Train	Train
191	kaempferide	COc1ccc(-c2oc3cc(O)cc(O)c3c(=O)c2O)cc1	3.75	Negative	Train	Train	Train	Train	Train	Hold-out test
192	xanthotoxol	O=c1ccc2cc3ccoc3c(O)c2o1	3.71	Negative	Train	Hold-out test	Train	Train	Train	Train
193	creatinine	CN1CC(=O)N=C1N	3.71	Negative	Train	Hold-out test	Train	Train	Train	Train
194	bergamottin	CC(C)=CCC/C(C)=C/COc1c2ccoc2cc2oc(=O)ccc12	4.01	Negative	Train	Train	Train	Train	Hold-out test	Train
195	alpelisib	Cc1nc(NC(=O)N2CCC[C@H]2C(N)=O)sc1-c1ccnc(C(C)(C)C(F)(F)F)c1	3.99	Negative	Hold-out test	Train	Train	Train	Train	Train
196	solriamfetol	NC(=O)OC[C@H](N)Cc1ccccc1	3.68	Negative	Train	Train	Train	Train	Train	Hold-out test
197	nialamide	O=C(CCNNC(=O)c1ccncc1)NCc1ccccc1	3.67	Negative	Hold-out test	Train	Train	Train	Train	Train
198	cerivastatin	COCc1c(C(C)C)nc(C(C)C)c(/C=C/[C@@H](O)C[C@@H](O)CC(=O)O)c1-c1ccc(F)cc1	3.67	Negative	Train	Train	Hold-out test	Train	Train	Train

199	stiripentol	CC(C)(C)C(O)/C=C/c1ccc2c(c1)OCO2	3.65	Negative	Train	Train	Train	Train	Hold-out test	Train
200	PROCAINAMIDE	CCN(CC)CCNC(=O)c1ccc(N)cc1	3.92	Negative	Hold-out test	Train	Train	Train	Train	Train
201	ciprofloxacin	O=C(O)c1cn(C2CC2)c2cc(N3CCNCC3)c(F)cc2c1=O	3.64	Negative	Train	Train	Train	Train	Train	Hold-out test
202	galangin	O=c1c(O)c(-c2ccccc2)oc2cc(O)cc(O)c12	3.63	Negative	Hold-out test	Train	Train	Train	Train	Train
203	TRICHLORMETHIAZIDE	NS(=O)(=O)c1cc2c(cc1Cl)NC(C(Cl)Cl)NS2(=O)=O	3.60	Negative	Train	Train	Train	Train	Train	Hold-out test
204	pramipexole	CCCN[C@H]1CCc2nc(N)sc2C1	3.85	Negative	Train	Train	Train	Hold-out test	Train	Train
205	VENLAFAXINE	COc1ccc(C(CN(C)C)C2(O)CCCCC2)cc1	3.53	Negative	Train	Train	Train	Hold-out test	Train	Train
206	monocrotaline	C[C@H]1C(=O)O[C@@H]2CCN3CC=C(COC(=O)[C@](C)(O)[C@]1(C)O)[C@H]23	3.45	Negative	Train	Train	Train	Train	Train	Hold-out test
207	anisodine	CN1[C@H]2CC(OC(=O)[C@@](O)(CO)c3ccccc3)C[C@@H]1[C@H]1O[C@@H]21	3.44	Negative	Train	Train	Train	Train	Hold-out test	Train
208	CETIRIZINE	O=C(O)COCCN1CCN(C(c2ccccc2)c2ccc(Cl)cc2)CC1	3.73	Negative	Train	Hold-out test	Train	Train	Train	Train
209	morin	O=c1c(O)c(-c2ccc(O)cc2O)oc2cc(O)cc(O)c12	3.35	Negative	Train	Train	Train	Train	Hold-out test	Train
210	R-atenolol	CC(C)NC[C@@H](O)COc1ccc(CC(N)=O)cc1	3.31	Negative	Train	Train	Train	Hold-out test	Train	Train
211	ombuin	COc1cc(O)c2c(=O)c(O)c(-c3ccc(OC)c(O)c3)oc2c1	3.30	Negative	Train	Train	Train	Train	Hold-out test	Train
212	TICLOPIDINE	Clc1ccccc1CN1CCc2sccc2C1	3.25	Negative	Hold-out test	Train	Train	Train	Train	Train
213	METFORMIN	CN(C)C(=N)N=C(N)N	3.32	Negative	Train	Train	Train	Train	Hold-out test	Train
214	S-atenolol	CC(C)NC[C@H](O)COc1ccc(CC(N)=O)cc1	3.19	Negative	Train	Train	Train	Train	Train	Hold-out test
215	histamine	NCCc1cnc[nH]1	3.12	Negative	Train	Train	Train	Hold-out test	Train	Train
216	topiramate	CC1(C)O[C@@H]2CO[C@@]3(COS(N)(=O)=O)OC(C)(C)O[C@H]3[C@@H]2O1	3.00	Negative	Hold-out test	Train	Train	Train	Train	Train
217	CAFFEINE	Cn1c(=O)c2c(ncn2C)n(C)c1=O	2.96	Negative	Train	Train	Hold-out test	Train	Train	Train
218	chloramphenicol	O=C(N[C@H](CO)[C@H](O)c1ccc([N+](=O)[O-])cc1)C(Cl)Cl	2.95	Negative	Train	Train	Train	Train	Hold-out test	Train
219	plazomicin	CN[C@@H]1[C@@H](O)[C@@H](O[C@@H]2[C@@H](O)[C@H](O[C@H]3OC(CNCCO)=CC[C@H]3N)[C@@H](N)C[C@H]2NC(=O)[C@@H](O)CCN)OC[C@]1(C)O	2.66	Negative	Train	Train	Train	Train	Hold-out test	Train
220	gentamicin	CNC(C)C1CCC(N)C(OC2C(N)CC(N)C(OC3OCC(C)(O)C(NC)C3O)C2O)O1	2.57	Negative	Hold-out test	Train	Train	Train	Train	Train
221	cephradine	CC1=C(C(=O)O)N2C(=O)[C@@H](NC(=O)[C@H](N)C3=CCC=CC3)[C@H]2SC1	2.39	Negative	Train	Train	Train	Train	Hold-out test	Train
222	amikacin	NCC[C@H](O)C(=O)N[C@@H]1C[C@H](N)[C@@H](O[C@H]2O[C@H](CN)[C@@H](O)[C@H](O)[C@H]2O)[C@H](O)[C@H]1O[C@H]1O[C@H](CO)[C@@H](O)[C@H](N)[C@H]1O	2.36	Negative	Train	Train	Train	Train	Train	Hold-out test
223	cefiderocol	CC(C)(O/N=C(\C(=O)N[C@@H]1C(=O)N2C(C(=O)[O-])=C(C[N+]3(CCNC(=O)c4ccc(O)c(O)c4Cl)CCCC3)CS[C@H]12)c1csc(N)n1)C(=O)O	2.33	Negative	Train	Train	Train	Train	Train	Hold-out test
224	tetramethylammonium	C[N+](C)(C)C	2.29	Negative	Train	Hold-out test	Train	Train	Train	Train
225	cephalexin	CC1=C(C(=O)O)N2C(=O)[C@@H](NC(=O)[C@H](N)c3ccccc3)[C@H]2SC1	2.19	Negative	Train	Hold-out test	Train	Train	Train	Train

Table S2 All metrics calculated for each model

Model	ROC-AUC	Accuracy	Precision	Sensitivity	Specificity	Youden's index	MCC	False rate	F-measure
MM-GB/SA regression	0.742 ± N.A.	0.684 ± N.A.	0.660 ± N.A.	0.614 ± N.A.	0.742 ± N.A.	0.356 ± N.A.	0.359 ± N.A.	0.316 ± N.A.	0.636 ± N.A.
RF (ECFP4)	0.824 ± 0.023	0.765 ± 0.018	0.786 ± 0.121	0.630 ± 0.039	0.876 ± 0.036	0.506 ± 0.065	0.516 ± 0.061	0.235 ± 0.018	0.697 ± 0.065
RF (ECFP4+MM-GB/SA)	0.833 ± 0.036	0.765 ± 0.047	0.779 ± 0.087	0.640 ± 0.114	0.862 ± 0.030	0.502 ± 0.104	0.512 ± 0.090	0.235 ± 0.047	0.698 ± 0.087
MPNN (Graph)	0.695 ± 0.096	0.684 ± 0.105	0.637 ± 0.140	0.716 ± 0.130	0.655 ± 0.146	0.371 ± 0.177	0.373 ± 0.181	0.316 ± 0.105	0.664 ± 0.118
MPNN (Graph+MM-GB/SA)	0.774 ± 0.023	0.698 ± 0.089	0.645 ± 0.135	0.714 ± 0.114	0.679 ± 0.104	0.393 ± 0.170	0.390 ± 0.174	0.302 ± 0.089	0.671 ± 0.113

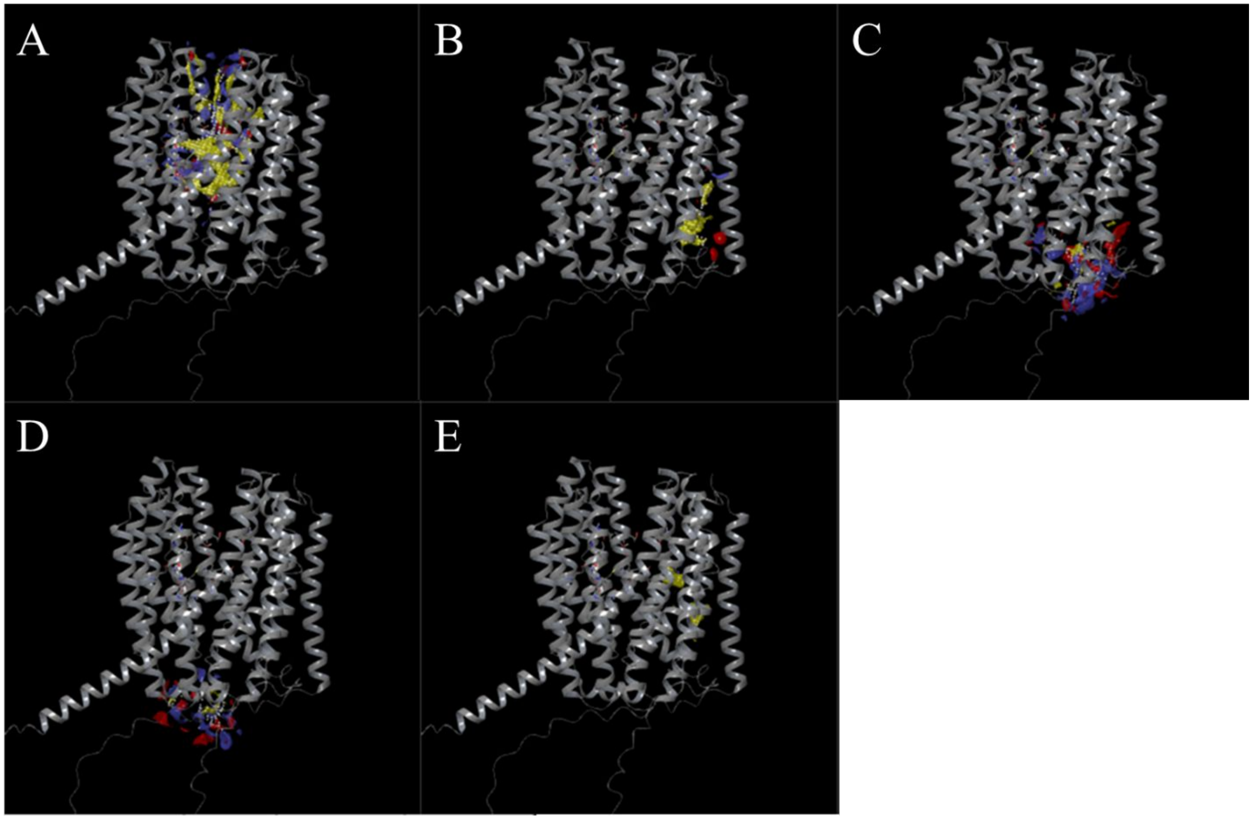

Figure S1 Prediction of Binding Sites

The SiteScores were (A) 1.191, (B) 1.145, (C) 1.035, (D) 0.899, and (E) 0.838. The best score was (A), and from the visual inspection the pathway of ligands is considered to be located in (A). Red region that prefers hydrogen bond acceptors; blue region that prefers hydrogen bond donors, and yellow region that prefers hydrophobicity.

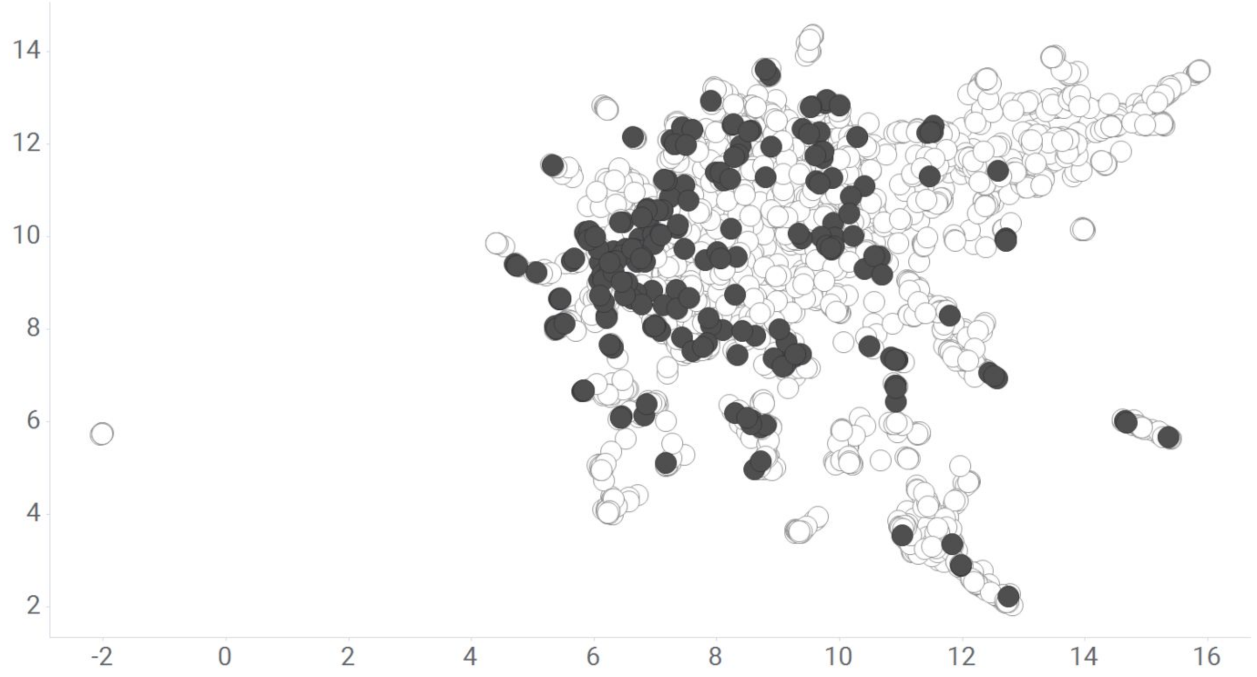

Figure S2 Chemical space analysis of the dataset used in this study and FDA approved compounds by UMAP

The x and y axis represents the score of first and second component in the UMAP analysis, respectively. The Black and white circle indicates the compounds used in this study for MATE inhibitory activity model and FDA approved drugs, respectively.

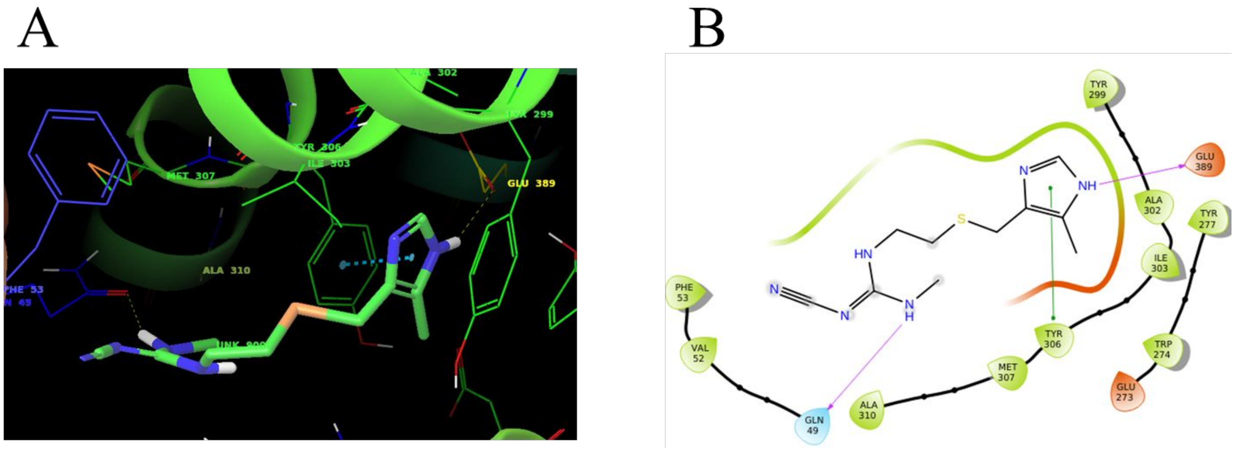

Figure S3 Docking Pose of Representative Drugs, cimetidine

The docking pose of cimetidine was observed in the Ligand Interaction Diagram of Maestro (Schrodinger Suite 2017). The docking pose and 2-dimensional interaction map of cimetidine are shown in A and B. In B, the color of amino-acids represents the properties such as green: hydrophobic, sky blue: polar, and red: negative charged. The straight line represents the interactions between cimetidine and amino-acids such as green: pi-pi stacking, and purple: H-bonding.

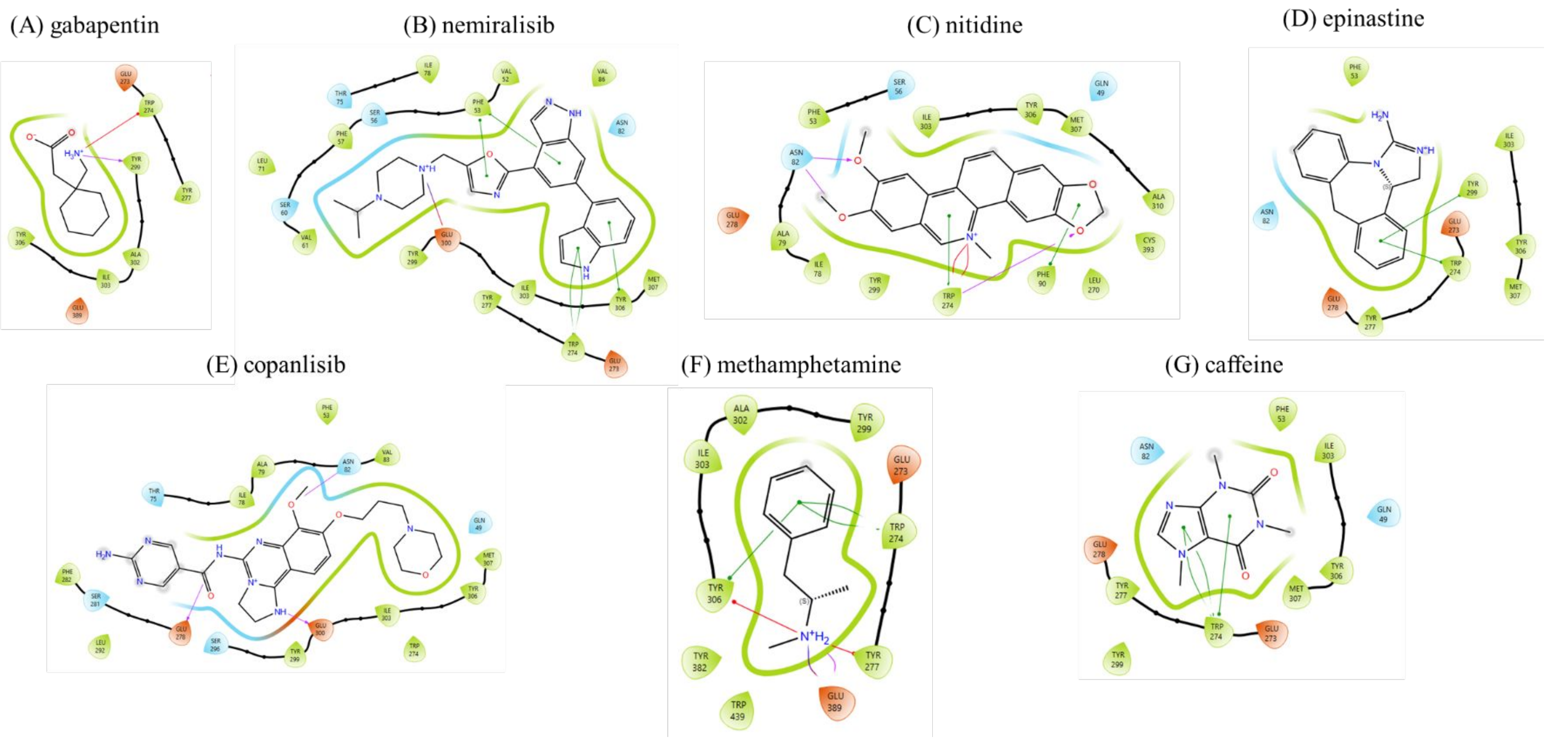

Figure S4 Picked Docking Poses of Inhibitors and Non-inhibitors

(A) gabapentin (pIC50: 8.96), (B) nemiralisib (pIC50: 7.59), (C) nitidine (pIC50: 6.82), (D) epinastine (pIC50: 5.96), (E) copanlisib (pIC50: 4.97), (F) methamphetamine (pIC50: 3.97), (G) caffeine (pIC50: 2.96). The amino-acids Gln49, Trp274, Tyr277, Tyr299, Ile303, Tyr306 were mostly contacted except for Gln49 to gabapentin, nemiralisib, epinastine, methamphetamine, and Try277 to nitidine,

copanlisib. The color of amino-acids represents the properties such as green: hydrophobic, sky blue: polar, and red: negative charged.

The straight line represents the interactions between cimetidine and amino-acids such as green: pi-pi stacking, and purple: H-bonding.
